# CuCo_2_S_4_ Nanosheets@N‐Doped Carbon Nanofibers by Sulfurization at Room Temperature as Bifunctional Electrocatalysts in Flexible Quasi‐Solid‐State Zn–Air Batteries

**DOI:** 10.1002/advs.201900628

**Published:** 2019-07-05

**Authors:** Zhenghui Pan, Hao Chen, Jie Yang, Yuanyuan Ma, Qichong Zhang, Zongkui Kou, Xiaoyu Ding, Yajun Pang, Lei Zhang, Qilin Gu, Chenglin Yan, John Wang

**Affiliations:** ^1^ Department of Materials Science and Engineering National University of Singapore Singapore 117574 Singapore; ^2^ School of Engineering Zhejiang A&F University Hangzhou 311300 P. R. China; ^3^ *i*‐Lab Suzhou Institute of Nano‐Tech and Nano‐Bionics Chinese Academy of Sciences Suzhou 215123 P. R. China; ^4^ College of Energy Soochow University Suzhou 215006 P. R. China

**Keywords:** bifunctional electrocatalysts, flexible, portable and wearable electronic devices, quasi‐solid‐state zinc–air batteries, room temperature

## Abstract

The performance of quasi‐solid‐state flexible zinc–air batteries (ZABs) is critically dependent on the advancement of air electrodes with outstanding bifunctional electrocatalysis for both the oxygen reduction reaction (ORR) and oxygen evolution reaction (OER), together with the desired mechanical flexibility and robustness. The currently available synthesis processes for high‐efficiency bifunctional bimetallic sulfide electrodes typically require high‐temperature hydrothermal or chemical vapor deposition, which is undesirable in terms of the complexity in experimental procedure and the damage of flexibility in the resultant electrode. Herein, a scalable fabrication process is reported by combining electrospinning with in situ sulfurization at room temperature to successfully obtain CuCo_2_S_4_ nanosheets@N‐doped carbon nanofiber (CuCo_2_S_4_ NSs@N‐CNFs) films, which show remarkable bifunctional catalytic performance (*E_j_*_= 10_ (OER) – *E*_1/2_ (ORR) = 0.751 V) with excellent mechanical flexibility. Furthermore, the CuCo_2_S_4_ NSs@N‐CNFs cathode delivers a high open‐circuit potential of 1.46 V, an outstanding specific capacity of 896 mA h g^−1^, when assembled into a quasi‐solid‐state flexible ZAB together with Zn NSs@carbon nanotubes (CNTs) film (electrodeposited Zn nanosheets on CNTs film) as the anode. The ZAB also shows a good flexibility and capacity stability with 93.62% capacity retention (bending 1000 cycles from 0° to 180°), making it an excellent power source for portable and wearable electronic devices.

## 1 Introduction

With the rapid development of portable and wearable electronic devices, there is an apparent demand for flexible energy storage devices, for which quasi‐solid‐state zinc–air batteries (ZABs) have acquired enormous attention because of their high theoretical energy density (1370 W h kg^−1^), abundant low‐cost resources for the starting materials required, and high device safety.^1^, ^2^, ^3^, ^4^, ^5^, ^6^, ^7^, ^8^ In principle, the advancement of flexible ZABs is critically dependent on the availability of desired air electrodes with excellent bifunctional electrocatalysis for both oxygen evolution reaction (OER) and oxygen reduction reaction (ORR), together with robust flexibility and mechanical strength.^9^, ^10^, ^11^, ^12^, ^13^, ^14^, ^15^, ^16^, ^17^, ^18^ For this purpose, several types of cathode materials have been explored. Bimetallic oxides (BOs)@heteroatom‐doped carbon hybrids have drawn considerable attentions, because of their high catalytic efficiency, derived from the synergetic effects between the nanostructured BOs (for OER) and N‐doping in carbon materials (for ORR).^19^, ^20^, ^21^, ^22^, ^23^, ^24^, ^25^, ^26^, ^27^ For example, CuCo_2_O_4_ quantum dots attached on N‐doped carbon nanotubes (CuCo_2_O_4_@NCNTs) were shown to be an impressively bifunctional electrocatalyst including a good positive half‐wave potential (0.80 V) for ORR and a low overpotential (467 mV@10 mA cm^−2^) for OER, which can contribute to an energy density of 659 W h kg^−1^ (in liquid state) for ZAB.^23^ Although great progress has been made recently with BOs@NC‐based ZABs in liquid state,^19^, ^20^, ^21^, ^22^, ^23^, ^24^, ^25^, ^26^, ^27^ the overall performances in quasi‐solid‐state are still far away from those key parameters satisfying the practical application requirement of ZABs in wearable electronics. The largely unsatisfactory performance in quasi‐solid‐state ZABs can be attributed to three main factors: i) limited catalytic activities arising from the sluggish ion/electron transport kinetic because of the relativity poor conductivity of BOs, especially in solid state electrolytes; ii) usage of polymer additives, such as polypyrrole^20^ and polyimide (PI),^24^ which are commonly employed to improve the flexibility of air electrodes, which however not only increase the “dead mass” for the electrodes and thus limit the energy density of the full devices, but also lead to the enlarged interfacial impedance with poor stability; iii) likely detachment of the weakly bound catalysts (BOs) from the N‐doped carbon materials, which have been commonly prepared by physical mixed methods, such as ball milling and blending, and often occur at the frequent and large deformation conditions.

In contrast to BOs (e.g., CuCo_2_O_4_), bimetallic sulfides (BSs, e.g., CuCo_2_S_4_) exhibit higher electrical conductivity and multiple valences endowing it with superior electrocatalyst activity.^28^, ^29^, ^30^ In addition, to properly make use of the intrinsic advantage of BSs, a novel fabrication approach is to directly grow the electrically conductive BSs on N‐doped carbon materials (BSs@NMSs), which would ensure with a better contact with the conductive matrix.^31^ In particular, it would be further desirable to develop the sulfide phase at a low temperature, ideally at room temperature. This will be in contrast to those processes completed at high hydrothermal temperatures^28^, ^29^, ^31^, ^32^ or chemical vapor deposition (CVD; they are usually higher than 600 °C),^33^, ^34^ which are tedious and time consuming. Most importantly, the mechanical strength of BSs@NCMs‐based electrodes is heat sensitive and easily damaged to lose flexibility in these processes,^31^, ^32^ thus also hinder its large‐scale application of flexible ZABs in portable and wearable electronic devices. Indeed, developing a low‐temperature processing strategy would be a quantum step forward for realizing a high surface area, large population of active sites, and retaining of the mechanical flexibility.^29^, ^35^, ^36^, ^37^

Herein, we describe a scalable fabrication process purposely developed for CuCo_2_S_4_ nanosheets@nitrogen‐doped carbon nanofibers (CuCo_2_S_4_ NSs@N‐CNFs) film with outstanding bifunctional electrocatalytic activity (*E_j_*_ = 10_ (OER) – *E*_1/2_ (ORR) = 0.751 V) and excellent mechanical flexibility, by effectively combining electrospinning with an in situ anion‐exchange process at room temperature. As a demonstration of being able to serve as an excellent cathode in metal–air batteries, the rechargeable flexible quasi‐solid‐state ZABs made of the CuCo_2_S_4_ NSs@N‐CNFs cathode are shown with a high open‐circuit potential of 1.46 V, excellent specific capacity of 896 mA h g^−1^, and an outstanding cycling stability, when it is assembled with Zn NSs@CNTs film (electrodeposited Zn nanosheets (NSs) on CNT film) as the anode in KOH gel electrolyte. They also demonstrate an exceptional flexibility and device stability with 93.62% capacity retention (bending 1000 cycles from 0° to 180°), which are highly desirable for use in portable and wearable electronics.

## 2 Results and Discussion

Among the various transition metal–based electrocatalysts, Co‐based nanomaterials have been considered to be one of the most promising candidates for the ORR/OER.^19^, ^38^, ^39^, ^40^, ^41^, ^42^ With the incorporation of Cu, the CuCo‐based electrocatalysts display a higher catalytic activity due to their tailored electron transfer between Cu and Co ions. As displayed in Figure S1a (Supporting Information), all diffraction peaks in the X‐ray diffraction (XRD) pattern of the as‐calcined sample are well indexed to cubic spinel‐type CuCo_2_O_4_ (PDF#78‐2177, space group: *Fd3m*), and refinement results present that the unit cell structure is composed of CuO_4_ tetrahedra and CoO_6_ octahedra (Figure S1b, Supporting Information, and which can be decomposed to CuO, Cu_2_O_3_, and Co_3_O_4_).^21^ Moreover, due to the solubility constants (*K*_sp_) of copper sulfide and cobalt sulfide are very low, 2.5 **×** 10^−48^ and 4 **×** 10^−21^, respectively, CuCo_2_O_4_ nanoparticles (NPs) have been successfully converted to CuCo_2_S_4_ NSs driven by the thermodynamic equilibrium in the Na_2_S solution (Figure S1c,d, Supporting Information).^28^, ^35^, ^36^ To further verify the concept of the in situ sulfurization at the room temperature, CuFe_2_O_4_ NPs (space group: *Fd3m*, and which can be decomposed to CuO and Fe_2_O_3_) and CoFe_2_O_4_ NPs (space group: *Fd3m*, stable cell structure) were chosen as the control samples. Encouragingly, the feasibility of the sulfurization at room temperature is also successfully demonstrated by the CuFe_2_O_4_ NPs sample, while there is no obvious change in the CoFe_2_O_4_ NPs (the detailed discussion can be seen in Figure S2 in the Supporting Information). These experimental results suggest that the room‐temperature in situ synthesis of BS_S_ NSs (CuCo_2_S_4_ NSs or CuFe_2_S_4_ NSs) may due to the high diffusion kinetics of S atoms, compared with those of O atoms in BOs’ lattices (the low *K*_sp_ of chalcogenides) and the incorporation of Cu ions in the spinel structured BOs (CuCo_2_O_4_ and CuFe_2_O_4_), where the anion exchange is facilitated between the transition‐metal chalcogenides and transition‐metal oxides.^28^, ^35^, ^36^

**Figure 1:**
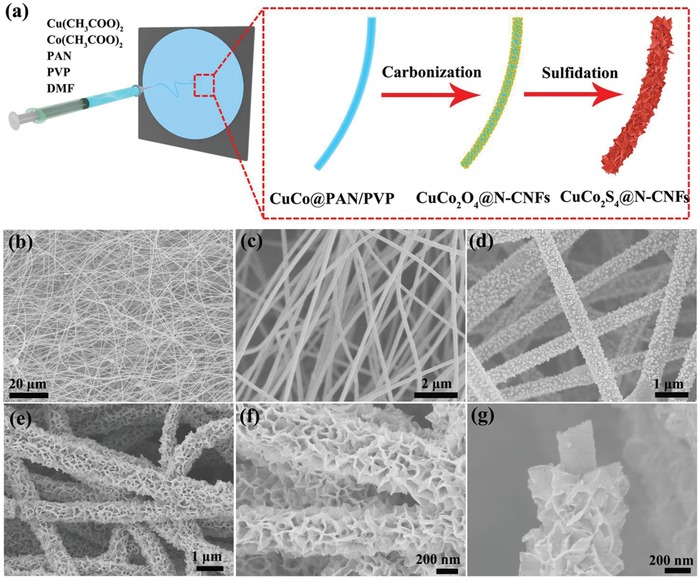
a) Schematic diagram illustration of the preparation processes for the CuCo_2_S_4_ NSs@N‐CNFs film. b,c) SEM images of the CuCo@PAN/PVP nanofibers with increasing magnifications. d) SEM image of CuCo_2_O_4_ NPs@N‐CNFs. e–g) SEM images of CuCo_2_S_4_ NSs@N‐CNFs with increasing magnifications.

**Figure 2:**
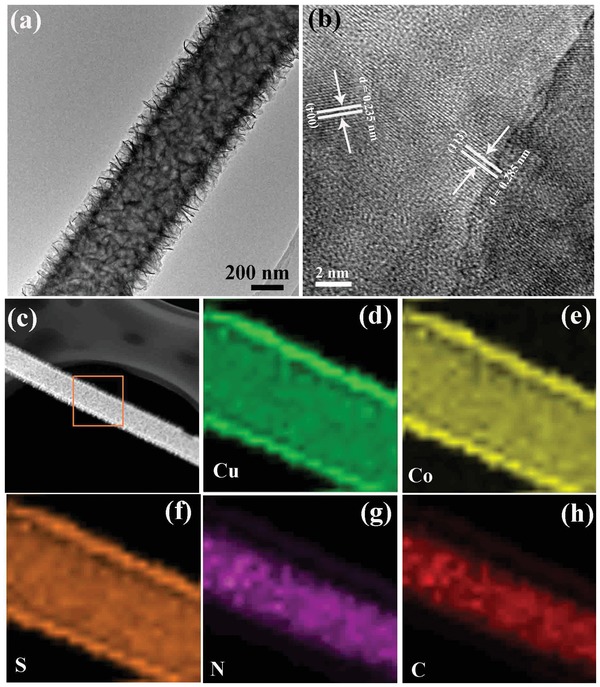
a) TEM image of the CuCo_2_S_4_ NSs@N‐CNFs. b) HRTEM image of the CuCo_2_S_4_ NSs shell. c–h) EDS mapping results for a single CuCo_2_S_4_ NSs@N‐CNF, demonstrating the CuCo_2_S_4_ NSs core and N‐CNF structure.

Based on the above experimental observations, the fabrication process of the CuCo_2_S_4_ NSs@N‐CNFs films is schematically illustrated in **Figure** **1**a, which involve electrospinning of N‐doped carbon fiber film, followed by carbonization/oxidation, and finally the in situ sulfurization of the CuCo_2_O_4_ NSs@N‐CNFs film (details are given in the “Experiment Section”). The CuCo_2_S_4_ NSs@N‐CNFs films thus developed have an outstanding tensile strength of 330 MPa (Figure S3, Supporting Information), which is of great importance for device stability of flexible ZABs.^4^, ^5^, ^6^ The morphology and nanostructure of the as‐obtained samples in every step were characterized by using scanning electron microscopy (SEM) and transmission electron microscopy (TEM). As presented in **Figure** **2**b,c and Figure S4 (Supporting Information), the electrospun fiber membrane is interwoven by numerous 1D nanofibers with the diameters of about 180–200 nm. The 1D fiber‐like nanostructure is well preserved after carbonization/oxidation at 400 °C (Figure 2d). From the high‐magnification SEM image (Figure S5, Supporting Information), exposed CuCo_2_O_4_ NPs are clearly observed on the surface of N‐CNFs, and no aggregation of NPs is found. The internal structure of CuCo_2_O_4_ NPs@N‐CNFs revealed by TEM studies further indicates that these NPs, of 30–100 nm in sizes, have grown rather uniformly on the N‐CNTs surface without aggregation (Figure S6, Supporting Information).

Figure 1e,f shows that the N‐CNFs were fully wrapped by the interconnected NSs, forming the CuCo_2_S_4_ NSs@N‐CNFs with core/shell structure (Figure 1g). The well‐distributed NSs on N‐CNF surface with a thickness of around 20 nm have effectively increased the active surface area of 853 m^2^ g^−1^ (371 m^2^ g^−1^ for CuCo_2_O_4_ NPs@N‐CNFs film; Figure S7, Supporting Information), thus contributing to improve the catalytic reaction between oxygen (O_2_), hydroxyl (OH^−^), and catalyst, and ensuring a good electrolyte accessibility.^43^, ^44^, ^45^ In Figure 2a, the TEM image shows the clear CuCo_2_S_4_ NSs@N‐CNFs core/shell structure. The high‐resolution TEM (HRTEM) edge view of CuCo_2_S_4_ discloses the distinct lattice spacings of 0.235 nm and 0.285 nm (Figure 2b), well matching up with the (004) and (113) planes of cubic CuCo_2_S_4_ phase, which is consistent with the XRD phase analysis results in Figure S2 (Supporting Information). From the high angle annular dark field (HAADF) image (Figure 2c) and energy dispersive spectroscopy (EDS) elemental mappings, one could note that Cu, Co, and S elements are distributed in the shell (Figure 2d,e), while N and C elements are located mainly in the core (Figure 2f,g). The elemental compositions of CuCo_2_S_4_ NSs@N‐CNFs are also confirmed by X‐ray photoelectron spectrum (XPS) analysis (Figure S8, Supporting Information). Moreover, the weight contents of CuCo_2_S_4_ and N‐CNFs were estimated to be 62.8% and 37.2% in the CuCo_2_S_4_ NSs@N‐CNFs structure from EDS, which is also supported by the thermogravimetric analysis (TGA) result (Figure S9, Supporting Information).

**Figure 3:**
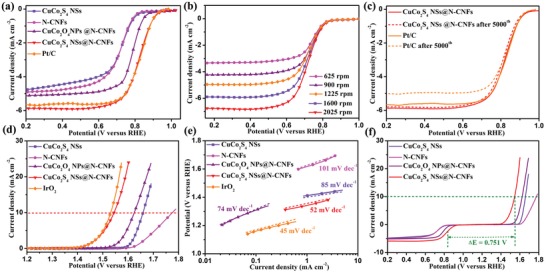
ORR polarization curves of a) the as‐prepared five samples, b) CuCo_2_S_4_ NSs@N‐CNFs at distinct rotation speeds, and c) the CuCo_2_S_4_ NSs@N‐CNFs and Pt/C electrodes before and after 5000th cycles. d) OER polarization curves, e) the corresponding Tafel plots, and f) ORR and OER polarization curves of the as‐prepared five samples.

Linear sweep voltammetry (LSV) and rotating ring‐disk electrode (RRDE) tests were performed in an O_2_‐saturated 0.1 m KOH electrolyte to investigate whether CuCo_2_S_4_ NSs@N‐CNFs could be acted as an extremely effective ORR/OER bifunctional electrocatalyst, with the CuCo_2_O_4_ NSs@N‐CNFs, N‐CNFs, CuCo_2_S_4_ NSs, and commercial Pt/C (20 wt%) being evaluated for comparison purposes. The CuCo_2_S_4_ NSs@N‐CNFs electrode shows an outstanding ORR activity including an onset potential of 0.957 V (vs reversible hydrogen electrode (RHE)), a half‐wave potential *E*_1/2_ of 0.821 V (vs RHE), and a limiting current density of 5.94 mA cm^−2^ at 0.2 V. These values are compared favorably with those of recently reported BSs‐based catalyst (**Figure** **3**a; Table S1, Supporting Information). Moreover, the CuCo_2_S_4_ NSs@N‐CNFs display the lowest Tafel slope of 51 mV dec^−1^ (Figure S10, Supporting Information), compared favorably to that of Pt/C (55 mV dec^−1^), which suggests its beneficial reaction kinetics. The outstanding catalytic activity toward ORR can be ascribed to the rationally designed CuCo_2_S_4_ NSs@N‐CNFs structure, which takes the full synergetic advantage of the high‐reactivity CuCo_2_S_4_ NSs and the rich N doping in N‐CNFs. Figure 3b presents the results of ORR LSV measurements for the CuCo_2_S_4_ NSs@N‐CNFs tested at different rotation speeds, and the homologous Koutechky–Levich (K‐L) plots are shown in Figure S11 (Supporting Information). The ideal linearity and persistent slope of the K‐L plots indicate the first‐order reaction to the dissolved O_2_ and analogous electron‐transfer number (*n*) during the ORR process. The electron transfer number per oxygen molecule (*n*) is ≈3.99 in the potential of 0.3–0.6 V, which is equivalent to the theoretical value of Pt/C (4.0). In addition, from the calculation of RRDE data, the HO_2_^−^ yield is below 9% at all potentials (Figure S12, Supporting Information). These results confirm that the CuCo_2_S_4_ NSs@N‐CNFs execute an ideal ORR activity by an efficient four‐electron pathway. Apart from the high activity, the CuCo_2_S_4_ NSs@N‐CNFs cathode also displays a considerable ORR stability after 5000 cycles, obviously outperforming than that of Pt/C (Figure 3c; Figure S13, Supporting Information).

The performance in OER activity is another important parameter for bifunctional electrocatalysts in rechargeable ZABs.^10^, ^45^ As expected, there is a lower overpotential (315 mV) required for CuCo_2_S_4_ NSs@N‐CNFs to reach a current density of 10 mA cm^−2^ (Figure 3d), which is outstandingly 81 mV lower than that of the CuCo_2_O_4_ NPs@N‐CNFs (396 mV) (Table S2, Supporting Information). The outstanding OER kinetics of CuCo_2_S_4_ NSs@N‐CNFs is also supported by its low Tafel slope of 48 mV dec^−1^, which is even comparable to that of commercial IrO_2_ (45 mV dec^−1^, Figure 3e). In general, the potential difference between OER and ORR (Δ*E* = *E_j_*_ = 10_ – *E*_1/2_) is employed to assess the overall activity of bifunctional catalytic performance, with the smaller value of Δ*E* implying better bifunctional activity.^46^, ^47^ Notably, the CuCo_2_S_4_ NSs@N‐CNFs exhibit a Δ*E* value of 0.751 V, which is the smallest value among the five samples investigated in the present work (Figure 3f), implying a high bifunctional activity. Note that the CuCo_2_S_4_ NSs or N‐CNFs electrodes present an obviously poorer ORR/OER catalytic performance and more sluggish catalytic kinetics, when compared with the CuCo_2_S_4_ NSs@N‐CNFs, which again demonstrate the synergetic effects between CuCo_2_S_4_ NSs and N‐CNFs. To better understand such synergetic effects, an electrochemical double layer capacitance (*C*_dl_) analysis was performed. As presented in Figure S14 (Supporting Information), the CuCo_2_S_4_ NSs@N‐CNFs delivers a *C*_dl_ of 35.13 mF cm^−2^, which is larger than that of CuCo_2_S_4_ NSs (6.95 mF cm^−2^) and N‐CNFs (8.42 mF cm^−2^), suggesting the largest electrochemical active surface area (EASA) for CuCo_2_S_4_ NSs@N‐CNFs electrode (the details can be seen in Figure S14 in the Supporting Information). The high EASA of CuCo_2_S_4_ NSs@N‐CNFs would provide a high specific surface area for the catalytically active sites and outstanding O_2_ bubble diffusion ability, thus resulting in an excellent catalytic performance. The superior ion and charge transport capabilities of CuCo_2_S_4_ NSs@N‐CNFs are demonstrated by the electrochemical impedance spectrum (EIS) studies shown in Figure S15 and Table S3 (Supporting Information).

**Figure 4:**
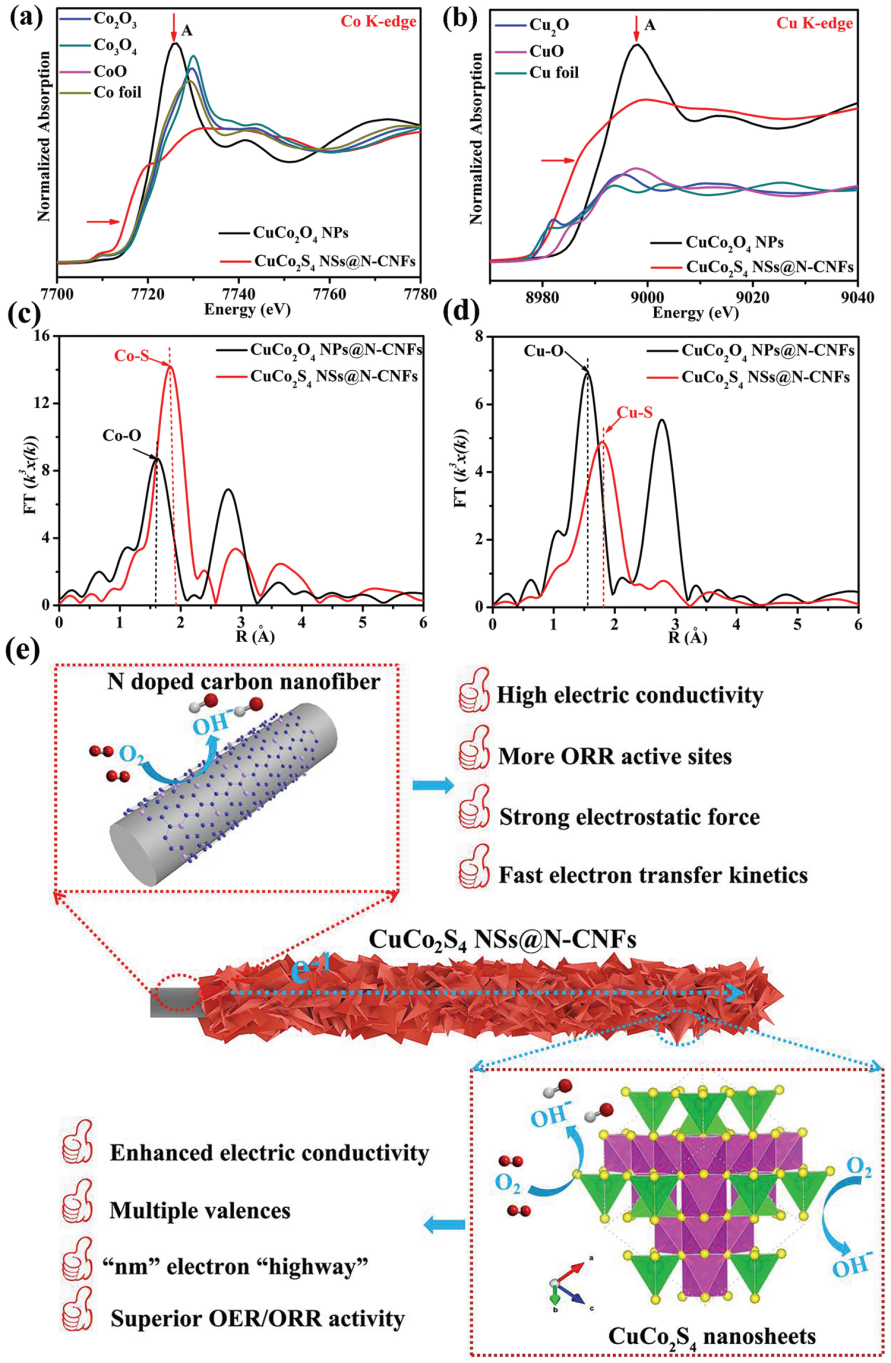
a) Normalized Co K‐edge XANES of CuCo_2_S_4_ NSs@N‐CNFs, CuCo_2_O_4_ NPs, Co_2_O_3_, Co_3_O_4_, CoO, and Co foil. b) Normalized Cu K‐edge XANES of CuCo_2_S_4_ NSs@N‐CNFs, CuCo_2_O_4_ NPs, Cu_2_O, CuO, and Cu foil. Fourier transform of the EXAFS data in *R*‐space of CuCo_2_S_4_ NSs@N‐CNFs for c) Co K‐edge and d) Cu K‐edge. e) Illustration of the catalytic process of CuCo_2_S_4_ NSs@N‐CNFs.

To better elucidate the catalytic mechanism behind the excellent ORR/OER performances of the CuCo_2_S_4_ NSs@N‐CNFs cathode, we carried out synchrotron X‐ray absorption near edge spectroscopy (XANES) to elucidate the electronic structure change of CuCo_2_S_4_ NSs in our unique core/shell electrode. As presented in **Figure** **4**a,b, comparing to the CuCo_2_O_4_ NPs powder sample, both the Co and Cu absorption edges in the XANES of CuCo_2_S_4_ NSs@N‐CNFs film show a left shift to lower energy and a lower peak A. These proofs demonstrate the presence of an interfacial interplay between N‐CNFs core and CuCo_2_S_4_ NSs shell. This interfacial interplay could manifestly promote the charge transport in CuCo_2_S_4_ NSs, and effect synergistically with the superior electrical conductivity N‐CNFs to further enhance the air cathode’s ORR/OER performances.^47^, ^48^ As a result, the highly conductive N‐CNFs film in our designed electrode not only guarantees the extraordinary flexibility for quasi‐solid‐state ZAB devices, but also provides the fast electron transfer kinetics for the CuCo_2_S_4_ NSs. This also well explains why the CuCo_2_S_4_ NSs@N‐CNFs film cathode shows a lower charge transfer resistance (*R*_ct_) than the CuCo_2_S_4_ NSs powder‐based cathode in Figure S15 (Supporting Information). Furthermore, Figure 4c shows the corresponding Fourier transform (FT) *k* ^3^χ(*k*) function of the extended X‐ray absorption fine structure (EXAFS) spectroscopy for CuCo_2_S_4_ NSs@N‐CNFs at Co K‐edge in *R‐*space. The dominant peaks at around 1.82 Å can be attributed to the Co—S bond, while the peak around 1.58 Å is related to the Co—O bond,^7^ confirming the successful sulfurization to CuCo_2_S_4_ NSs from CuCo_2_O_4_ NPs, which has also been confirmed by the EXAFS spectrum at Cu K‐edge in *R*‐space (Figure 4d).^19^, ^47^

As illustrated in Figure 4e, together with the XANES analyses and electrocatalytic results, the factors affecting the ORR/OER performances of CuCo_2_S_4_ NSs@N‐CNFs could be summarized as follows: 1) the highly nitridated N‐CNFs film with highly conductive and strong electrostatic force offers more ORR active sites (Figure S16, Supporting Information); 2) the intrinsic activity and electrical conductivity are both improved after in situ sulfurization from CuCo_2_O_4_ NPs to CuCo_2_S_4_ NSs, which leads to a superior electrocatalytic OER/ORR activity; 3) the nanosheets’ feature of multiple valances CuCo_2_S_4_ can be acted as a “nanometers highway” for electron transport; and 4) the large specific surface area originating from the CuCo_2_S_4_ NSs@N‐CNFs core/shell nanostructure. All of these advantages ensure our CuCo_2_S_4_ NSs@N‐CNFs film cathode with excellent ORR/OER activities.

**Figure 5:**
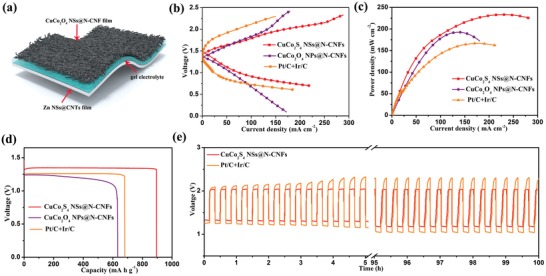
a) Schematic diagram illustration of the flexible quasi‐solid‐state ZABs. b) Charging–discharging polarization curves, c) power–current density curves, d) voltage–capacity curves, e) comparison of the cycling stabilities of the flexible ZABs.

Given the outstanding bifunctional ORR/OER catalytic activity and durability as discussed above, we assembled flexible quasi‐solid‐state ZABs using the CuCo_2_S_4_ NSs@N‐CNFs as the cathode and Zn NSs@CNTs film (electrodeposited Zn nanosheets on CNTs film) as anode (**Figure** **5**a; Figures S17 and S18, Supporting Information; and see the “Experimental Section” for details). The CuCo_2_S_4_ NSs@N‐CNFs‐based ZAB exhibits a high open‐circuit voltage (OCV) of 1.46 V, and the OCV can well be retained over 20 h (Figure S19, Supporting Information). The discharge–charge polarization curves of the ZAB is presented in Figure 5b, where one can see a lower voltage gap compared to that of Pt/C+Ir/C, implying a better rechargeability of the former. Moreover, a peak power density of 232 mW cm^−2^ (a maximum current density of 280 mA cm^−2^) was obtained from our ZABs (Figure 5c), much higher than those of CuCo_2_O_4_ NPs@N‐CNFs (192 mW cm^−2^, 180 mA cm^−2^) and Pt/C+Ir/C‐based ZABs (168 mW cm^−2^, 213 mA cm^−2^), and compared favorably with most of those reported works so far (Table S4, Supporting Information). Such outstanding performances of the ZAB with CuCo_2_S_4_ NSs@N‐CNFs as the cathode could well be correlated to the large Brunauer–Emmett–Teller (BET) area of CuCo_2_S_4_ NSs and the unique core@shell structure formed with N‐CNFs (Figure S8, Supporting Information), which endure an overall large amount of active sites and good O_2_ bubble diffusion ability. As shown in Figure 5d, the CuCo_2_S_4_ NSs@N‐CNFs‐based ZAB yields a specific capacity of 896 mA h g_Zn_^−1^ under a current density of 25 mA cm^−2^ (normalized to the total mass of Zn), corresponding to a large energy density of 965.2 W h kg^−1^ (Figure S20, Supporting Information). In addition, the cycling stability of ZAB was tested (10 min for charge and discharge in each cycle at 5 mA cm^−2^, Figure 5e; Figures S21 and S22, Supporting Information), where the ZAB with CuCo_2_S_4_ NSs@N‐CNFs cathode can be well stably cycled for a total of 300 cycles. With the above‐mentioned results, one can conclude that the CuCo_2_S_4_ NSs@N‐CNFs is a highly efficient air cathode, but much lower in cost compared with noble metals.

**Figure 6:**
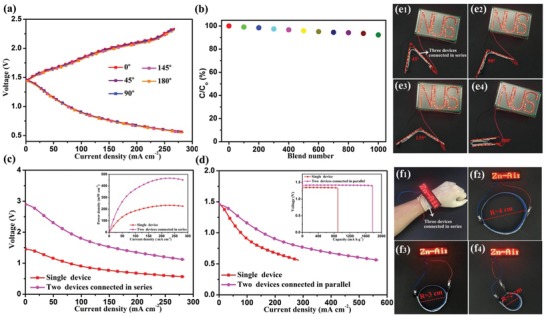
a) Discharge–charge polarization curves of the as‐fabricated flexible ZAB under different bending angles. b) Normalized capacity of the flexible ZABs bent 180° for 1000 cycles. Discharge polarization curves of the two flexible ZABs connected c) in series and d) in parallel. Digital optical images of three flexible ZABs connected in series to power a “NUS” logo with red LEDs under e1–e4) different bending angles, f1–f4) and a “Zn–air” breastpiece with red LEDs different bending radius.

As power sources for portable and wearable electronics, being able to tolerate harsh bending without remarkable loss in performance and reliability would be a requirement practical application of flexible ZABs. In this connection, the electrochemical performance of our flexible ZAB devices under different bending angles was evaluated. As shown in **Figure** **6**a, there is no apparent change that can be observed in the discharge–charge polarization curves at any given bending angles from 0° to 180° (Figure S23, Supporting Information), demonstrating that the ZAB devices were able to retain and thus possess the extraordinary flexibility. Furthermore, the specific capacity retention is still 93.62% even after bending 1000 cycles from 0° to 180° (Figure 6b), again showing the mechanical robustness of our ZAB device. The Nyquist plots of the flexible ZAB under different blending angles are presented in Figure S23b (Supporting Information), which demonstrates that the changes in charge transfer resistance (*R*_ct_) are negligible under the different blending conditions.

The working voltages reported among the best rechargeable ZABs are 1.15–1.35 V, which is much lower than that of commercial Li thin‐film batteries (3.5–4.0 V) and even less than that of Zn–MnO_2_ batteries (≈1.5 V).^1^, ^2^ The operating voltages of ZABs can be easily enhanced by integrating the devices in parallel or series to satisfy high‐voltage power and energy demands in practical applications. The quasi‐solid‐state and flexible properties of the ZAB devices are suitable for such designs, allowing for compatibility in available space of portable and wearable electronics. To evaluate the viability of our flexible ZABs, we have tested the energy and power performances of two ZAB devices connected in series and parallel. As shown in Figure 6c, the discharge voltage of two tandem devices almost doubled under the same discharge current compared with the single cell, implying a valid strategy to extend the output power with minimal power losses (see the inset in Figure 6c). In Figure 6d and its inset, the output current and capacity have also been increased by two times, when two ZAB devices are connected in parallel, again demonstrating the outstanding stability of the integrated ZAB devices. As a further application demonstration, the three tandem ZABs are capable of lighting a “NUS” logo with red light‐emitting diodes (LEDs, 1.8 V) under different bending angles (Figure 6e1–e4), highlighting their potential in wearable electronics. The three tandem ZABs can also be fabricated as a wearable bracelet and to power a “Zn–air” breastpiece with red LEDs on hand (Figure 6f1). The wearable bracelet can work perfectly even under different bending radius (Figure 6f2–f4), indicating outstanding flexibility and durability of our ZAB devices.

## 3 Conclusion

In summary, a scalable fabrication process for CuCo_2_S_4_ NSs@N‐CNFs films is successfully developed by effectively combining electrospinning with in situ sulfurization at room temperature. The CuCo_2_S_4_ NSs@N‐CNFs electrode thus developed can not only exhibit an outstanding bifunctional catalytic performance (*E_j_*_ = 10_ (OER) – *E*_1/2_ (ORR) = 0.751 V) in an alkaline medium, but also possess a high mechanical flexibility, thus being able to serve as a high‐performance cathode for flexible quasi‐solid‐state ZABs. The flexible ZAB assembled with CuCo_2_S_4_ NSs@N‐CNFs as the cathode and with Zn NSs@CNTs film as the anode delivers a high open‐circuit potential of 1.46 V, an outstanding specific capacity of 896 mA h g^−1^ (corresponding to a gravimetric energy densities of 965.2 W h kg^−1^), and an superior cycling stability. The excellent stability and flexibility of ZAB with 93.62% capacity retention (bending 1000 cycles from 0° to 180°) offer great application potential in rapidly emerging portable and wearable electronic devices. They can be integrated in parallel or in series to satisfy the power and energy demands in all kinds of applications, as demonstrated in the present work.

## 4 Experimental Section

*Preparation of CuCo_2_S_4_ NSs@N‐CNFs Film*: The CuCo_2_O_4_ NPs@N‐CNFs film was prepared by a simply electrospinning technique followed by carbonization/oxidation processes. Specifically, 1.6 g of polyacrylonitrile (PAN) and 0.2 g of polyvinylpyrrolidone (PVP) were dissolved in 10 mL *N*,*N*‐dimethylformamide (DMF) to obtain a polymer solution after stirring for 5 h to guarantee complete solubility (solution A). Meanwhile, 2.0 mmol Cu(CH_3_COO)_2_ and 4.0 mmol Co(CH_3_COO)_2_ were added in 10 mL DMF under continuous stirring for 5 h (solution B). Then, solution B was added to solution A, and the mixture was stirred for another 5 h to obtain working liquid for electrospinning. The precursor solution was transferred into a 6 mL syringe using an electrospinning setup and a 22 G × 1/2″ needle under working voltage between 12 and 13 kV. An aluminum foil was used as the receiving plate to collected electrospun nanofibers and then dried in vacuum oven at 70 °C for 12 h. To obtain CuCo_2_O_4_ NPs@N‐CNFs film, the as‐prepared nanofiber film was first stabilized in air at 270 °C for 2 h (1 °C min^−1^), followed by a carbonized at 400 °C for 1 h (1 °C min^−1^) under high‐purity N_2_ environment. Finally, the room‐temperature in situ sulfurization was performed by immersing the CuCo_2_O_4_ NPs@N‐CNFs film into 2.0 m Na_2_S solution for 1–2 h. The resulting CuCo_2_S_4_ NSs@N‐CNFs film was washed with ethanol and deionization (DI) water, and dried in vacuum oven at 70 °C for 24 h. For the control experiments, the CuCo_2_O_4_ NPs powder sample was first obtained by a simple hydrothermal process where the precursor solution contains 2.0 mmol Cu(CH_3_COO)_2_, 4.0 mmol Co(CH_3_COO)_2_, and 2.0 mmol NH_4_F, and then the CuCo_2_S_4_ NSs were obtained following the same steps as those used for the preparation of CuCo_2_S_4_ NSs@N‐CNFs film. Note that the CuFe_2_S_4_ NSs and CoFe_2_S_4_ NSs were also prepared by the same steps. In addition, the N‐CNFs film was also obtained by electrospinning technique without adding Cu(CH_3_COO)_2_ and Co(CH_3_COO)_2_ in the precursor solution.

*Electrodeposition of Zn Nanosheets on 3D CNTs’ Film (Zn NSs@CNTs Film)*: Zn was also deposited on CNTs’ film by an electrochemical deposition method. In brief, a two‐electrode system was used with a piece of CNTs’ film as work electrode (2 × 2 cm^2^) and a piece of Zn foil (2 × 2 cm^2^) as counter electrode. About 20 g of NaOH was dissolved in 200 mL DI water. Then, 1.6 g of ZnO was added to the mixture and stirred until complete dissolution. The obtained solution was selected as electrolyte. The electrodeposition was conducted with a constant voltage of −0.8 V for 30 min at room temperature.

*Assembly of the Flexible Quasi‐Solid‐State ZAB*: First, the KOH/polyvinyl alcohol (PVA) gel electrolyte was prepared by the previous works.^4^ Second, two pieces of CuCo_2_S_4_ NSs@N‐CNFs and Zn NSs@CNTs films were pressed on Au (100 nm)‐coated PI substrate (Au@PI). For the purpose of fabricating the quasi‐solid‐state device, the as‐obtained CuCo_2_S_4_ NSs@N‐CNFs electrode and Zn NSs@CNTs electrode were immersed into the gel electrolyte for 1 h, and subsequently remained at 70 °C for 24 h to evaporate the excess of water in the gel electrolyte. Then the quasi‐solid‐state ZAB was fabricated by pressing these two as‐prepared electrodes with gel electrolyte together under a pressure of ≈5 MPa for 10 min, which can promote the KOH/PVA gel electrolyte penetrating into each electrode and also form one thin separator layer.

## Conflict of Interest

The authors declare no conflict of interest.

## Supporting information

Supplementary
