# Supplementary material for: CuCo2S4 Nanosheets@N‐Doped Carbon Nanofibers by Sulfurization at Room Temperature as Bifunctional Electrocatalysts in Flexible Quasi‐Solid‐State Zn–Air Batteries
Source: Adv Sci (Weinh). 2019 Jul 5;6(17):1900628. doi: 10.1002/advs.201900628 (PMC6724571; doi:10.1002/advs.201900628)
Supplement: Supplementary file 1 — Supplementary [file ADVS-6-1900628-s001.pdf]

Copyright WILEY-VCH Verlag GmbH & Co. KGaA, 69469 Weinheim, Germany, 2019.

ADVANCED SCIENCE
Open Access

Supporting Information

for *Adv. Sci.*, DOI: 10.1002/advs.201900628

$CuCo_2S_4$ Nanosheets@N-Doped Carbon Nanofibers by Sulfurization at Room Temperature as Bifunctional Electrocatalysts in Flexible Quasi-Solid-State Zn–Air Batteries

Zhenghui Pan, Hao Chen, Jie Yang, Yuanyuan Ma, Qichong Zhang, Zongkui Kou, Xiaoyu Ding, Yajun Pang, Lei Zhang, Qilin Gu, Chenglin Yan,* and John Wang**

Supporting Information

$CuCo_2S_4$ Nanosheets@N-doped Carbon Nanofibers by Sulfurization at Room Temperature as Bifunctional Electrocatalysts in Flexible Quasi-solid-state Zn-Air Batteries

Zhenghui Pan, Hao Chen,* Jie Yang, Yuanyuan Ma, Qichong Zhang, Zongkui Kou, Xiaoyu Ding, Yajun Pang, Lei Zhang, Qilin Gu, Chenlin Yan,* and John Wang*

*Corresponding Author: msewangj@nus.edu.sg; haochen@zafu.edu.cn; c.yan@suda.edu.cn

Materials characterizations

Field-emission Scanning Electron Microscopy (SEM) was performed with a Zeiss Supra 40VP operating from 5.0-20.0 kV. Transmission Electron Microscopy (TEM) was conducted using a JEOL-2010F at an accelerating voltage of 200 kV. Aberration corrected STEM was carried out with a JEM-ARM200F equiped with a cold field emission gun and an ASCOR corrector operating at 200 kV. X-ray Photoelectron Spectroscopy (XPS) was carried out using a Kratos Axis Ultra DLD and all data were referenced to the C 1s = 284.8 eV. X-Ray Powder Diffraction (XRD) was carried out with Bruker D8 diffractometer, respectively. ICP-OES was performed using the Thermo Scientific iCAP 6300. The tensile properties were measured using an universal material testing machine (Instron 3365, Instron Corporation, USA) at a cross-head speed of 2 mm/min. XANES was conducted at the Singapore Synchrotron Light Source (SSLS), XAFCA beamline.

Electrochemical measurement

A three-electrode system was used to test the activities of $CuCo_2S_4$ NSs@N-CNFs in 0.1 M KOH electrolyte which is saturated and protected by bubbling O_2 with a flow of 20 sccm, where a Ag/AgCl and a Pt net as reference electrodes and counter electrode, respectively. All electrochemical measurements were carried out at 25 ^oC. For OER measurement, the $CuCo_2S_4$ NSs@N-CNFs (2 × 2 cm^2) were directly used as the working electrodes. The $CuCo_2S_4$ NSs@N-CNFs were attached to a glassy carbon electrode (5 mm diameter) and then used as the working electrodes in the ORR measurement. The scan rate for ORR/OER measurements was 5 mV s^{-1}. The ORR polarization curves were collected at various rotation rates ranging from 625 to 2025 rpm for calculating the transferring electron number. The accelerated durability tests were performed at room temperature in O_2 saturated 0.1 M KOH solution at a sweep rate of 50 mV s^{-1}

for 3000 cycles.

For flexible all-solid-state ZAB, polarization curves were obtained from galvanodynamic measurements (potentiostat, Autolab PGSTAT302N). Electrochemical impedance spectroscopy (EIS) measurements were carried out by applying an AC voltage with 20 mV amplitude in a frequency range from 17 kHz to 1 Hz at 0.8 V. Cycling test was performed using recurrent galvanostatic pulses for 10 min of discharge followed by 10 min of charge at 1 mA cm^{-2}.

Calculation of electron transfer number (n) and HO_2^- (%) for oxygen reduction reaction

On the basis of RDE data, Koutecky-Levich plots are generated by plotting the inverse of diffusion limiting current, i_{dl}^{-1} as a function of one over square root of rotating speed, $\omega^{-1/2}$ (rad s^{-1}). The number of electron transferred during ORR, *n* (i.e., electron transfer number) can be extracted from the slope of Koutecky-Levich plots on the basis of Koutecky-Levich equation

$$\frac{1}{i} = \frac{1}{i_k} + \frac{1}{i_{dl}} = \frac{1}{i_k} + \frac{1}{0.62nFAC_{O_2}D_{O_2}^{2/3}v^{-1/6}w^{1/2}} \quad (1)$$

$$\text{slope} = (0.62nFAC_{O_2}D_{O_2}^{2/3}v^{-1/6}w^{1/2})^{-1} \quad (2)$$

where *F* is Faraday constant (96485 C mol^{-1}), *A* is the geometric area of the electrode (0.25 cm^2), and *v* is the kinematic viscosity of electrolyte. D_{O_2} is the diffusion coefficient of oxygen in the electrolyte, while C_{O_2} is the concentration of the dissolved oxygen in the electrolyte at partial pressure of 1 atm. D_{O_2}, C_{O_2}, and *v* in 1 M KOH is about 1.9×10^{-5} cm^2 s^{-1}, 1.2×10^{-7} mol cm^{-3}, and 0.01 cm^2 s^{-1}, respectively.

For the RRDE measurements, the disk electrode was scanned at a rate of 5 mV s^{-1}, and the ring potential was constant at 1.3 V vs. RHE. The % HO_2^- and transferred electron number per oxygen molecule (n) were determined by the followed equations:

$$HO_2^- = 200\frac{I_r/N}{I_d+I_r/N} \quad (3)$$

$$n = 4 \frac{I_r / N}{I_d + I_r / N} \quad (4)$$

where I_d is disk current, I_r is ring current, and N is current collection efficiency of the Pt ring. N was determined to be 0.40.

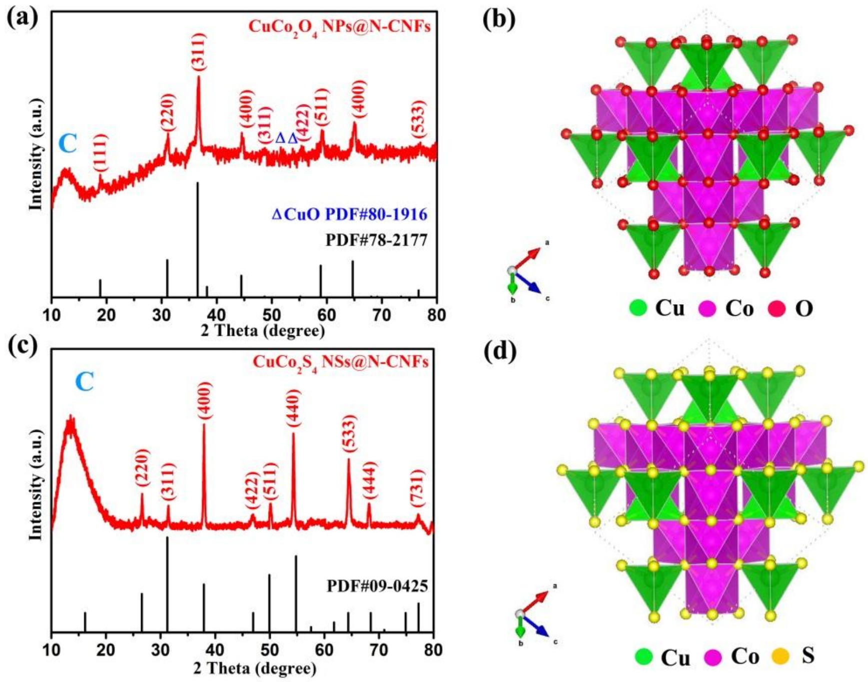

Figure S1. (a) XRD pattern of $CuCo_2O_4$ NPs@N-CNFs. (b) Crystal structure consisting of CuO_4 tetrahedra (green) and CoO_6 octahedra (pink). (c) XRD pattern of $CuCo_2S_4$ NSs@N-CNFs. (d) Crystal structure consisting of CuS_4 tetrahedra (green) and CoS_6 octahedra (pink).

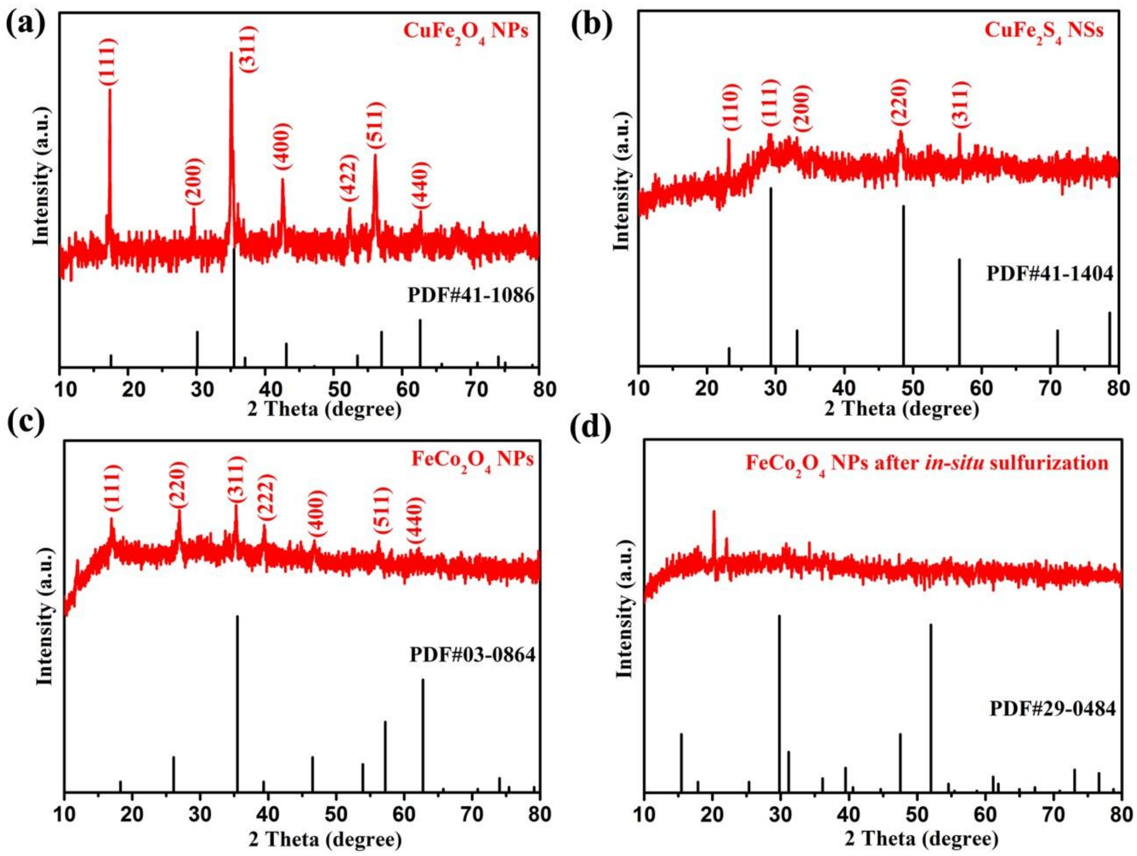

Figure S2. XRD patterns of $CuFe_2O_4$ NPs (a), $CuFe_2S_4$ NSs (b), $FeCo_2O_4$ NPs (c) amd $FeCo_2O_4$ NPs after *in-situ* sulfurization.

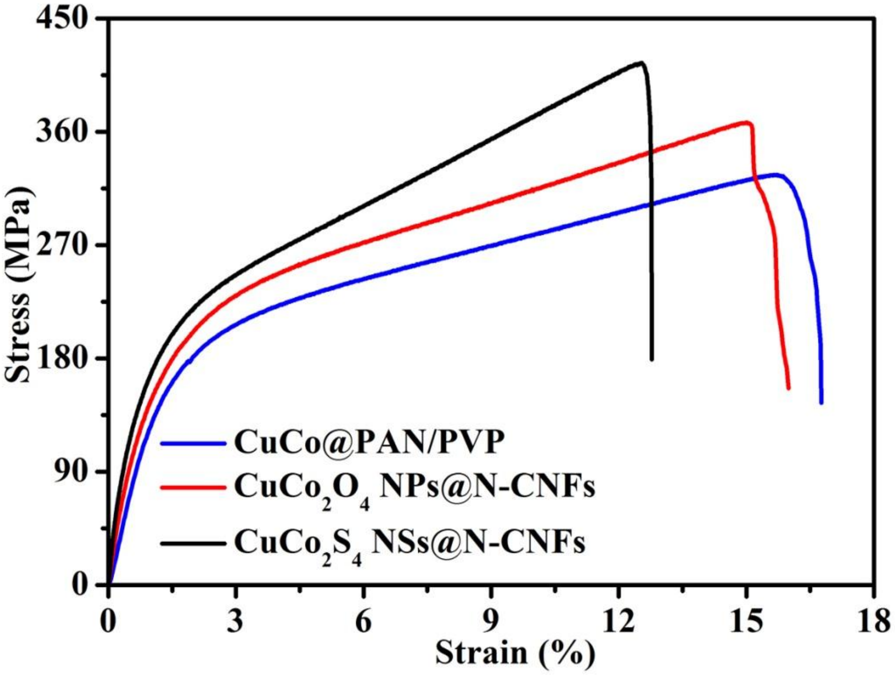

Figure S3. Comparison of stress-strain curves of pristine CuCo@PAN/PVP film, $CuCo_2O_4$ NPs@N-CNTs film and $CuCo_2S_4$ NSs@N-CNTs film.

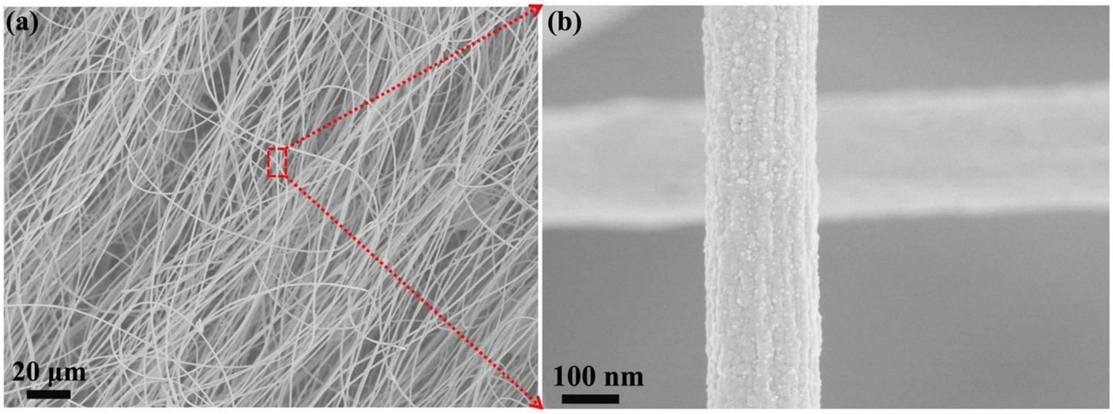

Figure S4. SEM images of the as-spun nanofibers with increasing magnifications.

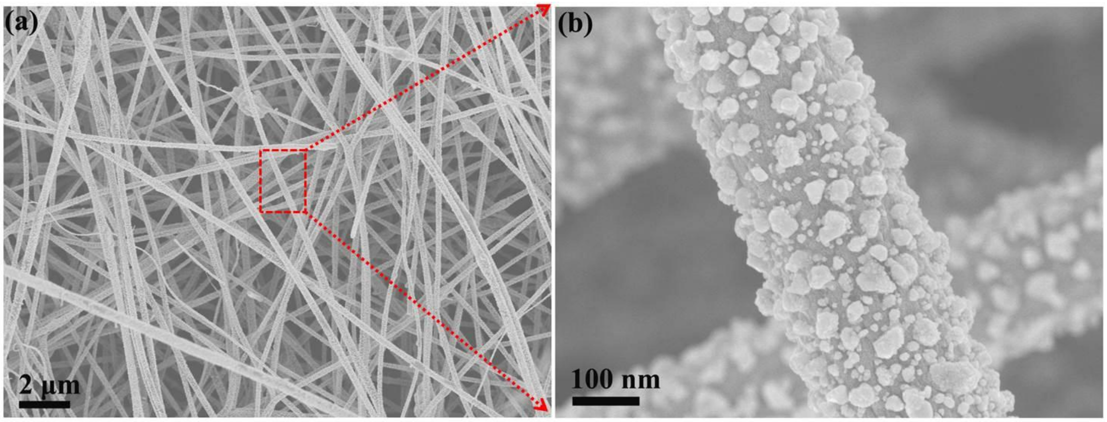

Figure S5. (a, b) SEM images of $CuCo_2O_4$ NPs@N-CNFs film at increasing magnification.

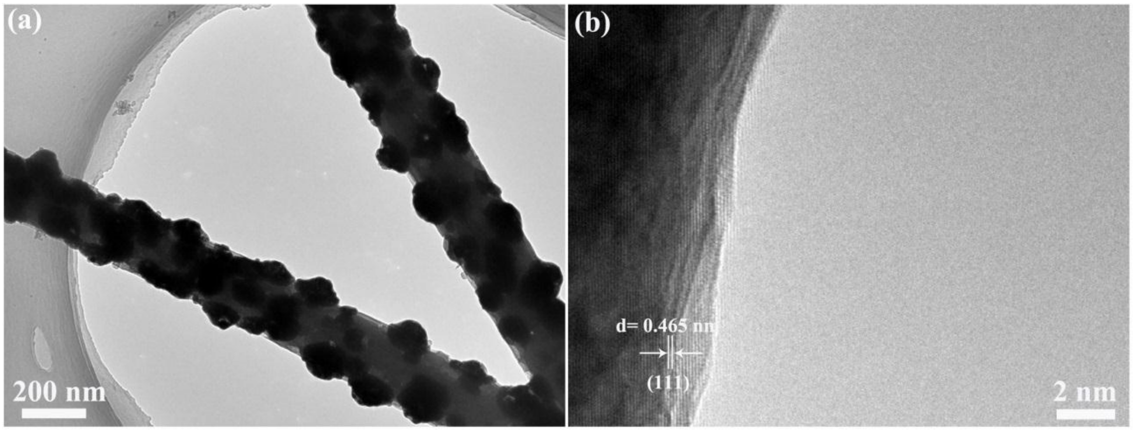

Figure S6. (a) TEM image of the $CuCo_2O_4$ NPs@N-CNFs. (b) HRTEM image of the $CuCo_2O_4$ NPs.

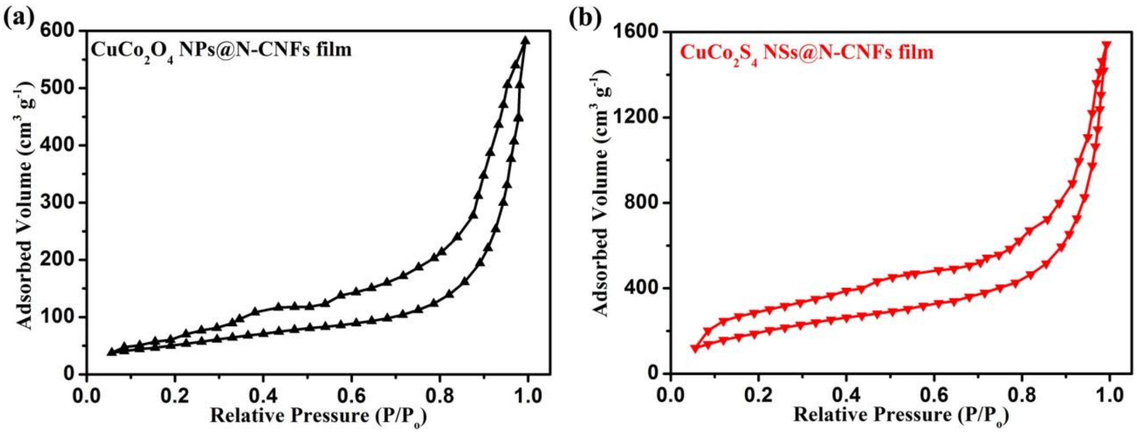

Figure S7. Nitrogen adsorption and desorption isotherms of the $CuCo_2O_4$ NPs@N-CNFs film (a) and $CuCo_2S_4$ NSs@N-CNFs film (b).

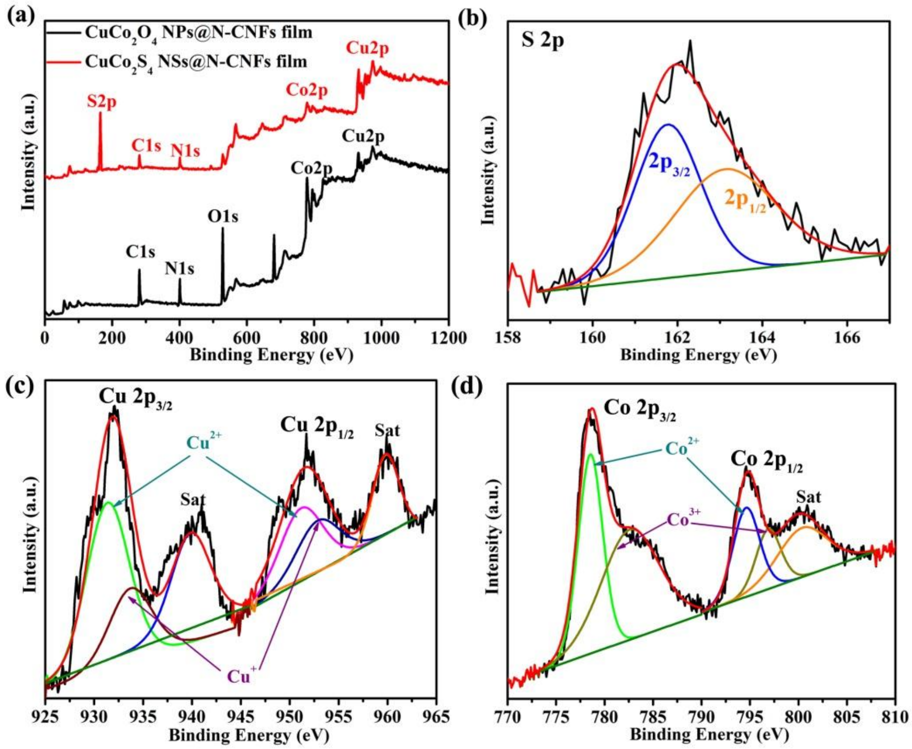

Figure S8. (a) XPS spectra of the $CuCo_2O_4$ NPs@N-CNFs film and $CuCo_2S_4$ NSs@N-CNFs film. High-resolution XPS of (b) S 2p, (c) Cu 2p, and f) Co 2p, respectively.

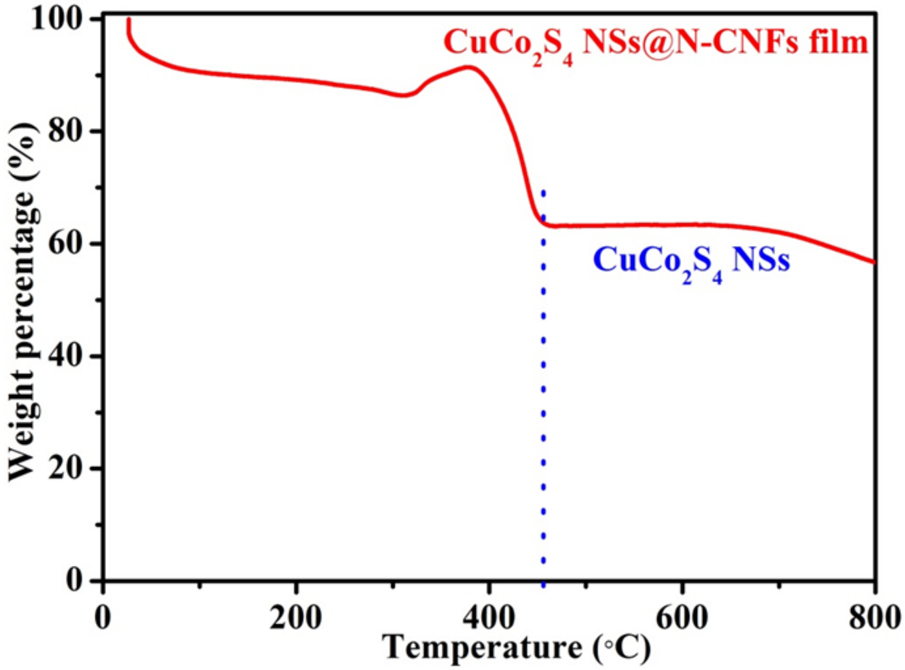

Figure S9. TGA trace of $CuCo_2S_4$ NSs@N-CNFs film.

Table S1 A comparison of the electrocatalytic activity of recently reported highly active ORR oxygen catalysts.

Catalysts	ORR				Refs
	Onset pontential (V)	Half-wave potential (V)	Limited diffusion current density (mA)	Tafel slope mV decade^{-1}	
**$CuCo_2S_4$ NSs@N-CNFs**	**0.957**	**0.821**	**-5.94**	**51**	**This work**
Pt/C	0.982	0.829	-5.59	55	
NC-Co_3O_4-90	0.911	NA	-12.82	NA	1
Co_3O_4@N-CNT aerogel	0.90	0.81	-5.3	84	2
N_2-Co_3O_4	0.94	NA	NA	57.6	3
Ultrathin Co_3O_4@CC	NA	NA	NA	NA	4
Co_3O_4@N-rGO	0.90	0.79	-5.34	54	5
Co@Co_3O_4@NC	0.92	0.8	-4.72	NA	6
Co_3O_4@N-rmGO	NA	0.83	-5.0	42	7
FeN_x-embedded PNC	0.98	0.83	-4.84	NA	8
$Fe_{0.5}Co_{0.5}O_x$@NrGO-300	0.90	NA	-5.25	62.2	9
NiFe@NG	0.98	0.83	-5.0	NA	10
NGM-Co	0.79	NA	-4.75	58	11
$CuCoO_x$@C	0.951	NA	NA	NA	12
NiFe@NC_x	1.03	0.86	-5.85	64.1	13
$Pb_2Ru_2O_{6.5}$	0.89	0.81	-5.7	60	14
NiFe-LDH/Co,N-CNF	0.893	0.79	-5.0	NA	15
FeCo@N-GCNT	1.03	0.92	-5.4	66.8	16
CuS/NiS_2 INs	0.89	0.73	-4.67	79	17
NiS_2/CoS_2-O NWs	0.85	0.70	NA	NA	18
CoN_4/NG	0.98	0.87	NA	70	19
Co@NG	0.68	NA	-4	NA	20
$CuCo_2O_4$/N-CNTs	NA	NA	-5.53	76.53	21
Co-N_x/C NA	0.91	0.877	-5.1	66	22
C-MOF-C2-900	NA	0.817	-5.980	NA	23
Co_2P nanocrystals	0.86	0.858	-4.64	72.1	24

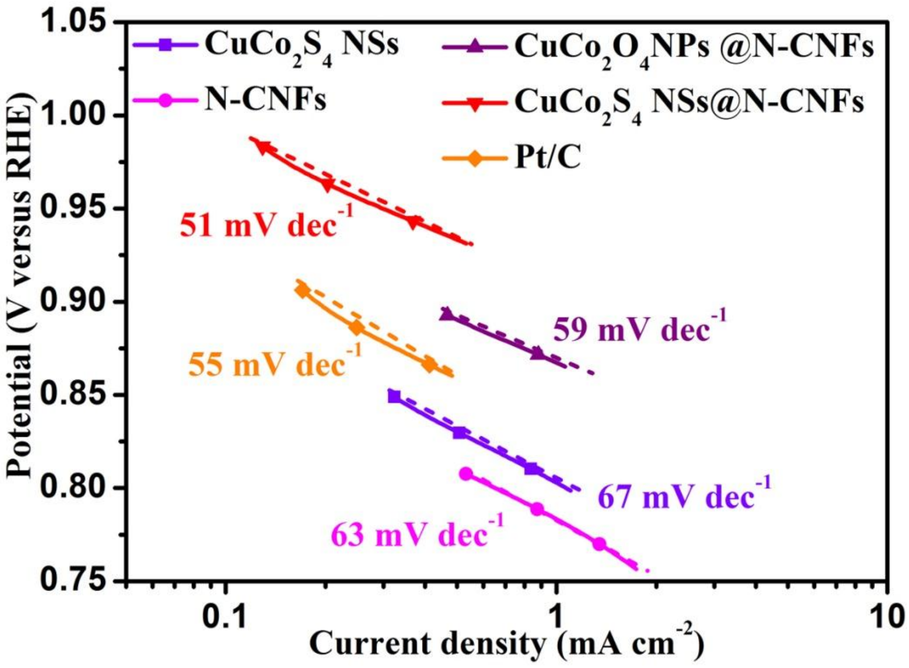

Figure S10. The corresponding Tafel plots of ORR polarization curves.

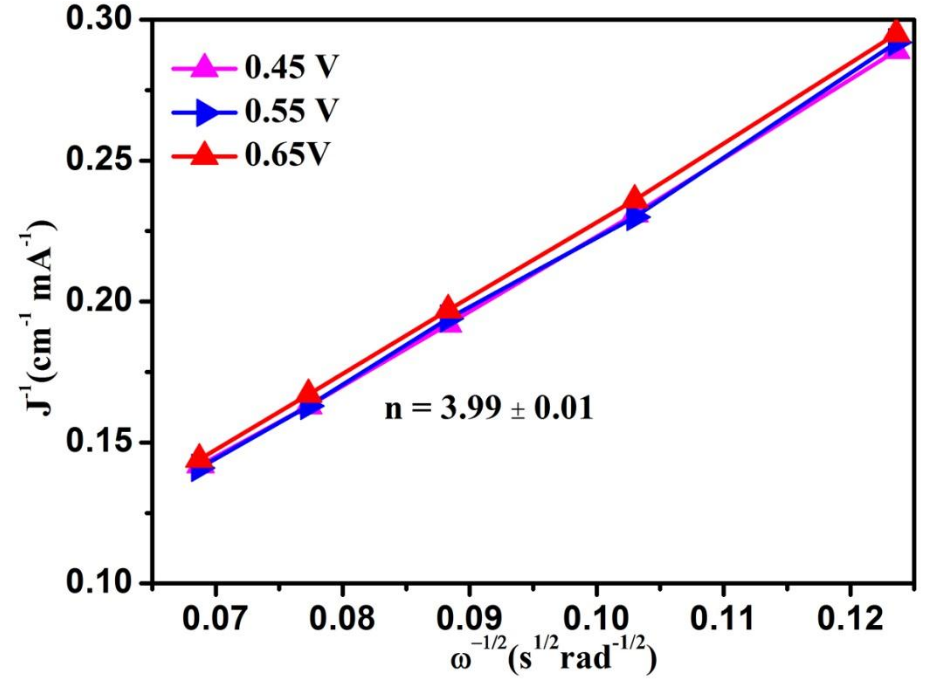

Figure S11. The Koutecky-Levich plots of the $CuCo_2S_4$ NSs@N-CNFs, showing that the calculated electron transfer number is ≈3.99.

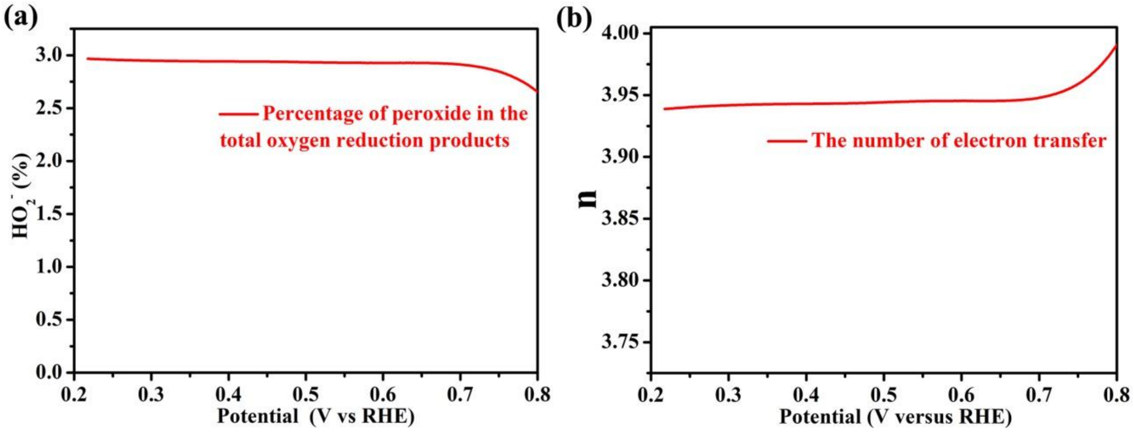

Figure S12. Percentage of peroxide in the total oxygen reduction products (a) and the number of electron transfer (b) at the $CuCo_2S_4$ NSs@N-CNFs electrode and Pt/C electrodes based on the RRDE result.

Figure S12a shows the percentage of peroxide species with respect to the total oxygen reduction products, and Figure S12b shows the electron transfer numbers calculated from the RRDE curves. It can be envisioned that oxygen molecules were reduced to water via a nearly four-electron pathway (*n* is over 3.94) with a small ratio of peroxide species (less than 3.0%), in agreement with the results from the K-L plots.

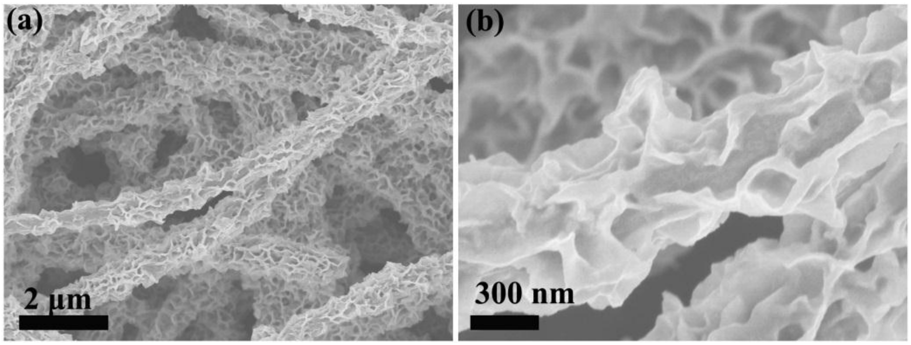

Figure S13. (a, b) SEM images of the $CuCo_2S_4$ NSs@N-CNFs electrode after 5000 cyclings in the ORR test.

Table S2 A comparison of the electrocatalytic activity of recently reported highly active OER oxygen catalysts.

Catalysts	OER			
	Onset pontential (V)	Overpotential (mV, 10 mA cm^{-2})	Tafel slope mV $decade^{-1}$	Refs
**$CuCo_2S_4$ NSs@N-CNFs**	**1.41**	**315**	**48**	**This work**
$Fe_{0.5}Co_{0.5}O_x$@NrGO-300	NA	257	30.1	9
NiS_2/CoS_2-O NWs	1.44	235	31	18
CuS/NiS_2 INs	1.59	290	36	17
NC-Co_3O_4-90	NA	358	NA	1
Co_3O_4@N-CNT aerogel	1.45	470	NA	2
Ultrathin Co_3O_4@CC	NA	NA	NA	4
Co_3O_4@N-rGO	1.53	490	101	5
Co@Co_3O_4@NC	1.50	370	94	6
Co_3O_4@N-rmGO	1.51	310	67	7
FeN_x-embedded PNC	1.58	390	80	8
NiFe@NG	1.60	380	115	10
NGM-Co	NA	NA	NA	11
$CuCoO_x$@C	NA	327	74	12
NiFe@NC_x	1.46	320	60.6	13
$Pb_2Ru_2O_{6.5}$	1.48	410	114.2	14
NiFe-LDH/Co,N-CNF	1.5	310	78	15
FeCo@N-GCNT	1.41	500	99.5	16
$CuCo_2O_4$/N-CNTs	NA	NA	118.8	21
Co-N_x/C NA	NA	300	62.3	22
C-MOF-C2-900	1.52	350	79	23
Co_2P nanocrystals	NA	330	NA	24

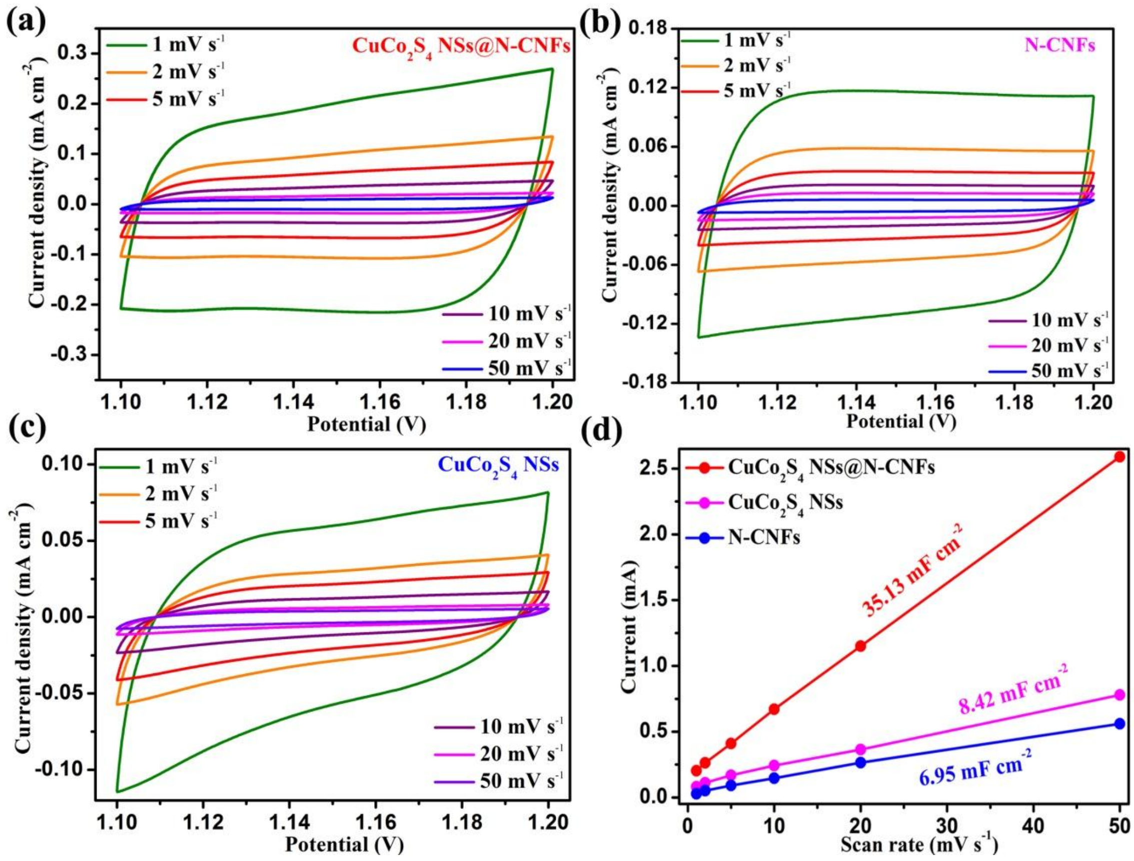

Figure S14. CVs of (a) $CuCo_2S_4$ NSs@N-CNFs, (b) $CuCo_2S_4$ NSs and (c) N-CNFs at scan rates from 1 to 50 mV s^{-1}. (d) Scan rate vs the current of the three electrodes.

We have also carried out the electrochemical active surface area (EASA) from electrochemical double layer capacitance (C_{dl}) by using CV to investigate the reasons behinds of the superior performance of $CuCo_2S_4$ NSs@N-CNFs better than that of N-CNFs and $CuCo_2S_4$ NSs. As shown in Figure S14 (d), a linear fit determined the specific capacitance to be 35.13 mF cm^{-2} for $CuCo_2S_4$ NSs@N-CNFs, 8.16 mF cm^{-2} for N-CNFs and 6.95 mF cm^{-2} for $CuCo_2S_4$ NSs, suggesting the largest EASA for $CuCo_2S_4$ NSs@N-CNFs electrode. The current measured from CV were linearly fitted with CV scan rates using the following equation: $i = v\ C_{dl}$, where v is the scan rate and i is the current. C_{dl} value can be obtained from the slope of the above equation.

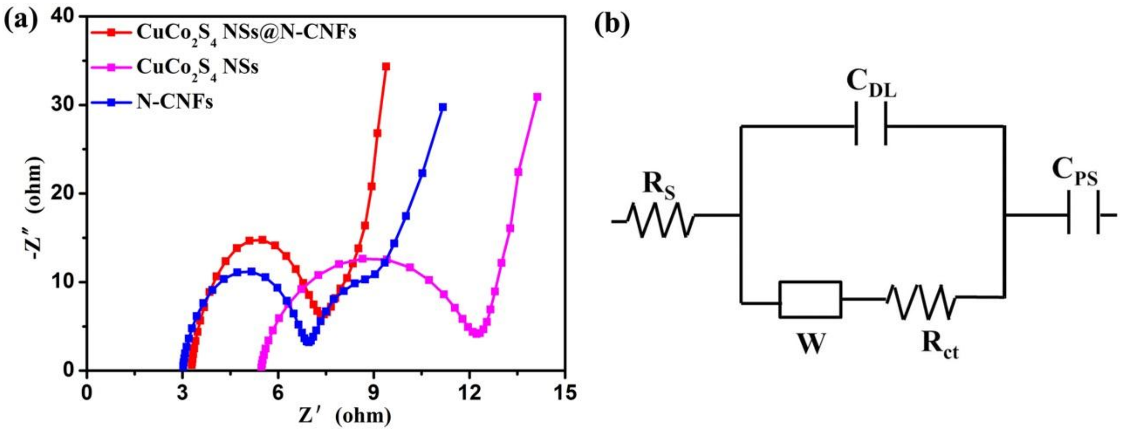

Figure S15. (a) The EIS of Nyquist plots of $CuCo_2S_4$ NSs@N-CNFs, $CuCo_2S_4$ NSs and N-CNFs, respectively, and the coresspongding equivalent circuit (b).

As shown in Figure S15(a), the impedance data were analyzed by fitting to an equivalent circuit consisting of the series resistance (R_s), charge transfer resistance (R_{ct}), double layer capacitance (C_{DL}) and Warburg behavior (W). At high frequency range, the value of the intercept at the real axis is used to estimate the R_s of the electrodes, which is the combination of the ionic resistance of the electrolyte and the resistance of the electrode material itself. The semicircle diameter in the plot corresponds to the R_{ct}, also called Faraday resistance, corresponding to the total resistance at the interface between the electrode and the electrolyte.

Table S3. Fitting results for Electrochemical Impedance Spectroscopy

Electrodes	R_s (Ω)	R_{ct} (Ω)	C_{DL}	W
$CuCo_2S_4$ NSs@N-CNFs	3.28	4.07	0.78794	0.69874
$CuCo_2S_4$ NSs	5.47	6.87	0.76524	0.67895
N-CNFs	3.02	3.96	0.77426	0.69941

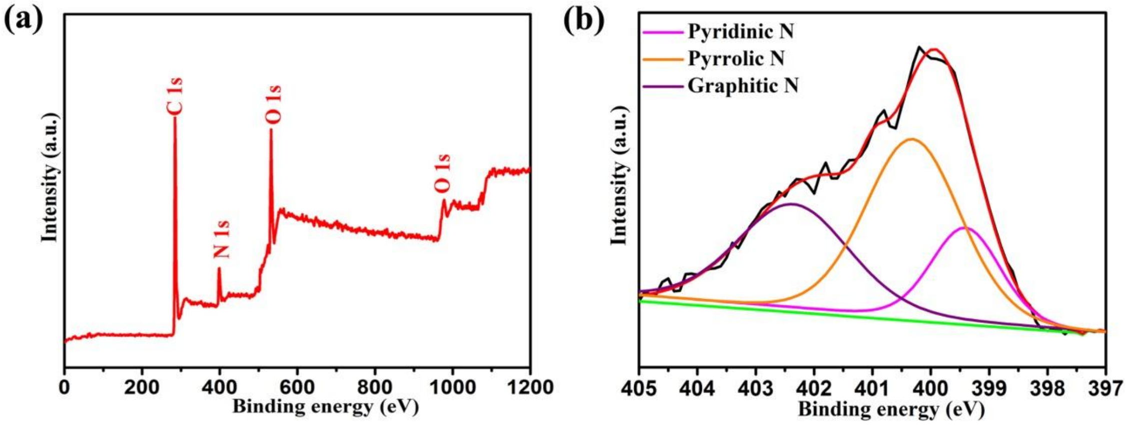

Figure S16. XPS spectra of N-CNFs film (a) survey spectrum, and (b) N 1s.

As presented in Figure S16a, The survey XPS spectrum clearly revealed that the product contains C (72.3 at%), N (15.35 at%), and O (12.35 at%) elements. The high resolution N 1s spectra can be assigned to three components including pyridinic N, pyrrolic N and graphitic N (Figure S16b). It is well known that doping sp^2 carbon with heteroatoms, such as N, and O, could not only enhance the bulk electric conductivity but also increase the wettability of the carbon framework.

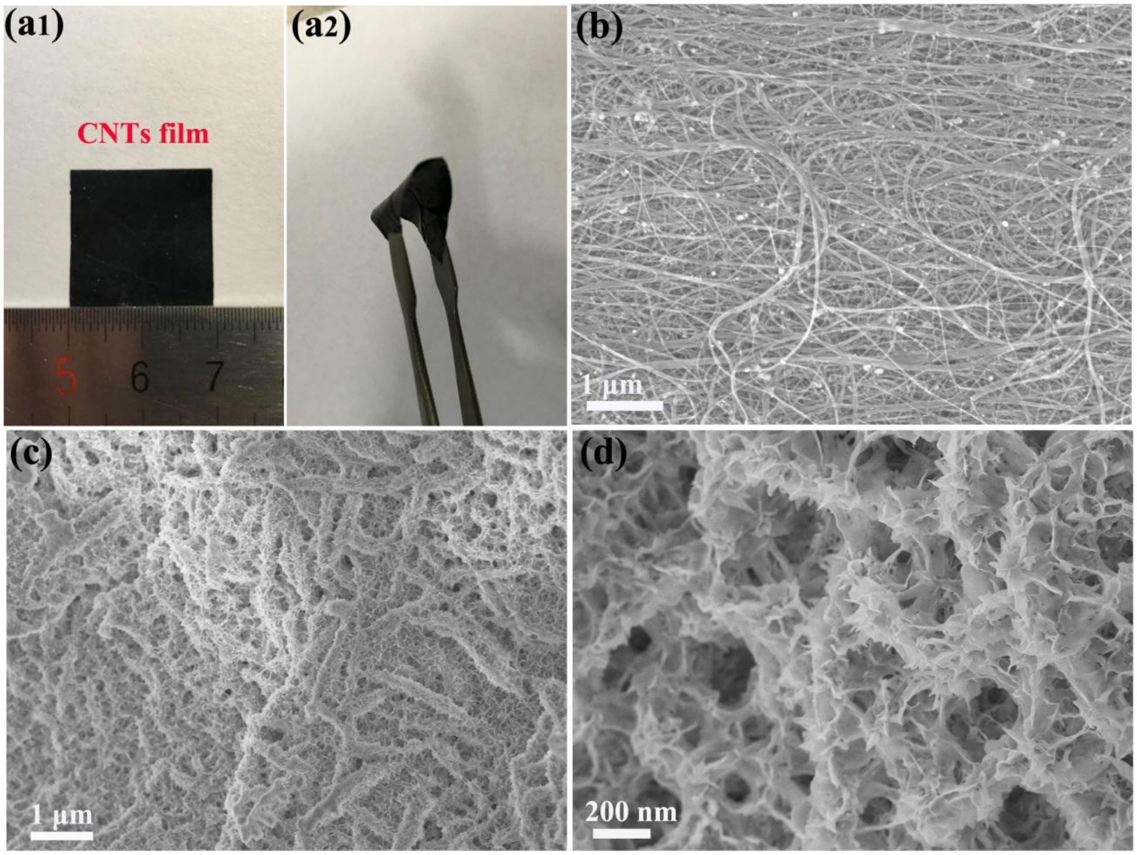

Figure S17. (a) Optical image and (b) SEM image of CNTs film. (c, d) SEM images of Zn NSs@CNTs film.

As the negative electrode of the flexible all-solid-state ZAB, Zn nanosheets were uniformly deposited on the surface of the CNTs film by electrochemical deposition (Figure S17). The crystal structure of Zn was characterized by XRD, as shown in Figure S18a, where all of the peaks correspond to hexagonal zinc (JCPDS # 87-0713). Furthermore, the Zn nanosheets were further confirmed to be in the metallic state by the XPS results shown in Figure S18b.

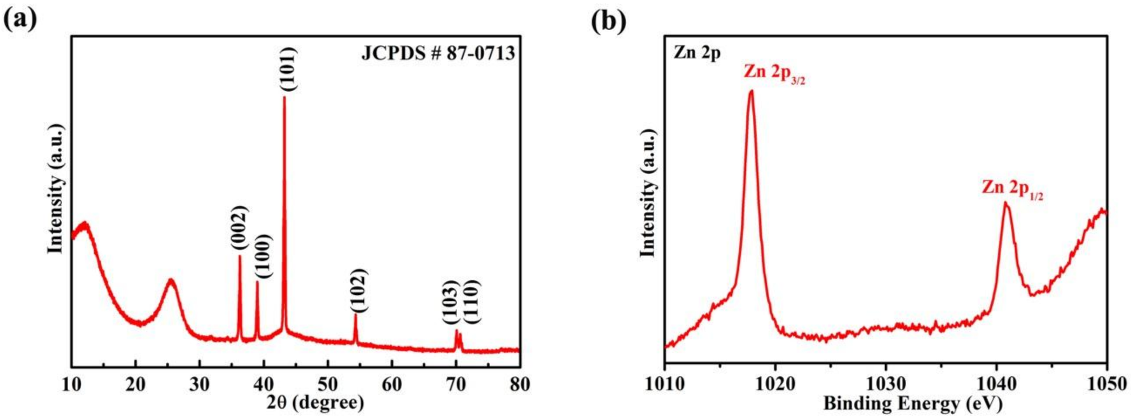

Figure S18. (a) XRD pattern, and (b) XPS spectrum of Zn NSs@CNTs film.

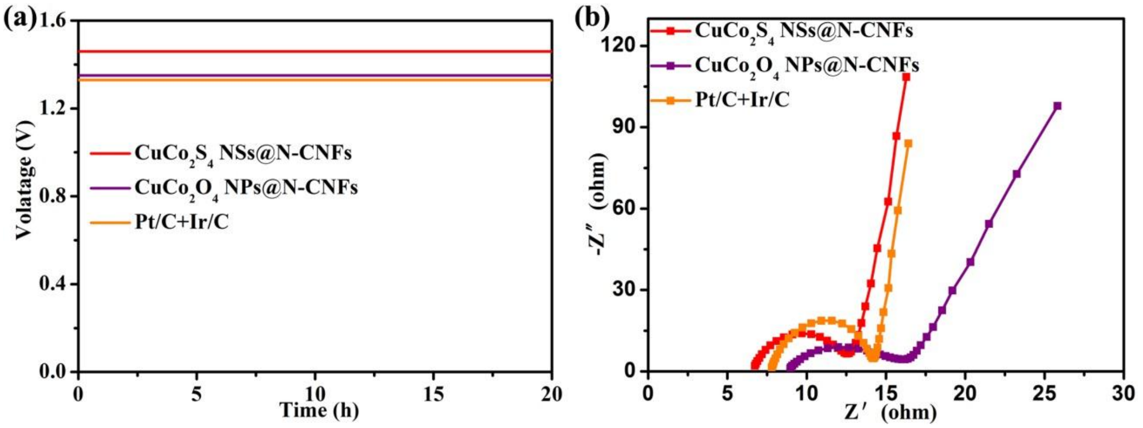

Figure S19. (a) Open-circuit plots and (b) EIS spectra of ZABs based on $CuCo_2S_4$ NSs@N-CNFs, $CuCo_2O_4$ NPs@N-CNFs and Pt/C+Ir/C electrodes as air cathode.

Table S4 Summarized properties of the recently reported solid-state ZAB devices

Types of device	Open circuit potential (V)	Capacity (mAh g^{-1})	Maximum current density (mA cm^{-2})	Peak power density (mW cm^{-2})	Refs
**$CuCo_2S_4$ NSs@N-CNFs**	**1.46**	**896 (25 mA cm^{-3})**	**280**	**232**	**This work**
NC-Co_3O_4/CC	1.44	387.2 (25 mA cm^{-3})	227	82 (mW cm^{-3})	1
Co_3O_4@N-CNT aerogel	1.31	NA	NA	NA	2
N-Co_3O_4/CC	1.1	603.7 (2.5 mA cm^{-3})	NA	32 (mW cm^{-3})	3
Ultrathin Co_3O_4@CC	1.33	495 (6 mA cm^{-3})	NA	NA	4
Co_3O_4@N-rGO	1.20	550 (6 mA cm^{-3})	NA	NA	5
Co@Co_3O_4@NC	1.228	685 (5 mA cm^{-3})	NA	64	6
FeN_x-embedded PNC	1.55	NA	195	278	8
FeNi@NG	1.2	NA	NA	80.8	10
NGM-Co	1.44	749.4 (20 mA cm^{-3})	NA	152	11
$Pb_2Ru_2O_{6.5}$	1.23	NA	NA	195	14
NiFe-LDH/Co,N-CNF	1.50	NA	NA	NA	15
CuS/NiS_2 INs	1.44	775	NA	172.4	17
NiS_2/CoS_2-O NWs	1.49	NA	NA	265	18
CoO/N-CNT	1.40	570	NA	350	19
Co@NG	1.40	NA	NA	NA	20
Co-N_x/C NA	1.42	853.12	NA	NA	22
C-MOF-C2-900	1.46	768	NA	NA	23
Co_2P nanocrystals	1.34	NA	NA	61	24
MnO_x@GCC	1.427	NA	NA	32	25
$ZnCo_2O_4$ QD@NC	1.47	428.47	150	82.3	26
$Fe_{0.5}Co_{0.5}O_x$@NrGO-300	1.43	756 (10 mA cm^{-3})	NA	86	9
$CuCoO_x$@C	1.41	NA	NA	NA	12
$CuCo_2O_4$/N-CNTs	1.36	817.4	200	83.8	21
FeCo@N-GCNT	1.48	872.2	NA	89.3	16
NiFe@NC_x	1.35	583.7	NA	NA	13
$CuCo_2S_4$ NSs	1.20	241	NA	NA	27
NiO/CoN PINWs	1.46	648	200	79.6	28
$NiCo_2S_4$/S-GNS	1.38	NA	375	216	29
MnO-$(MnFe)_2O_3$/NCNT	1.45	647	155	100	30

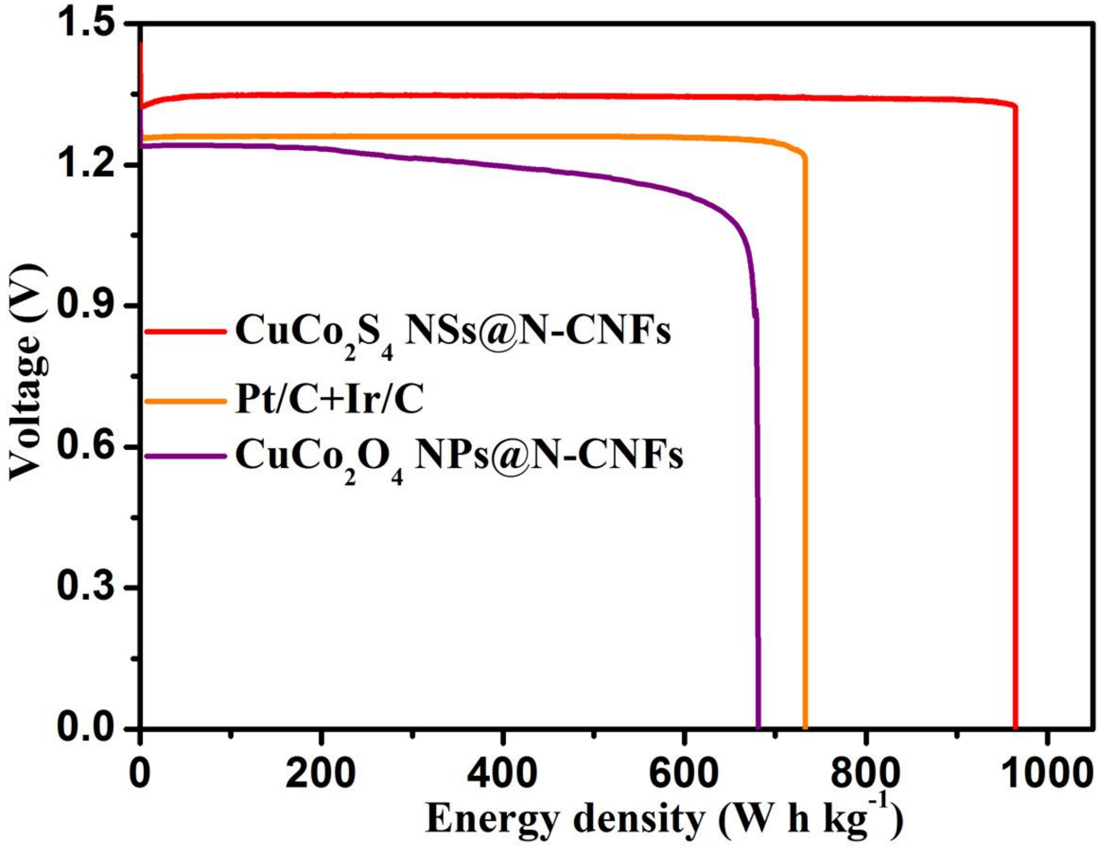

Figure S20. Energy density plots of ZABs based on $CuCo_2S_4$ NSs@N-CNFs, $CuCo_2O_4$ NPs@N-CNFs and Pt/C+Ir/C electrodes as air cathode at 25 mA cm^{-2}.

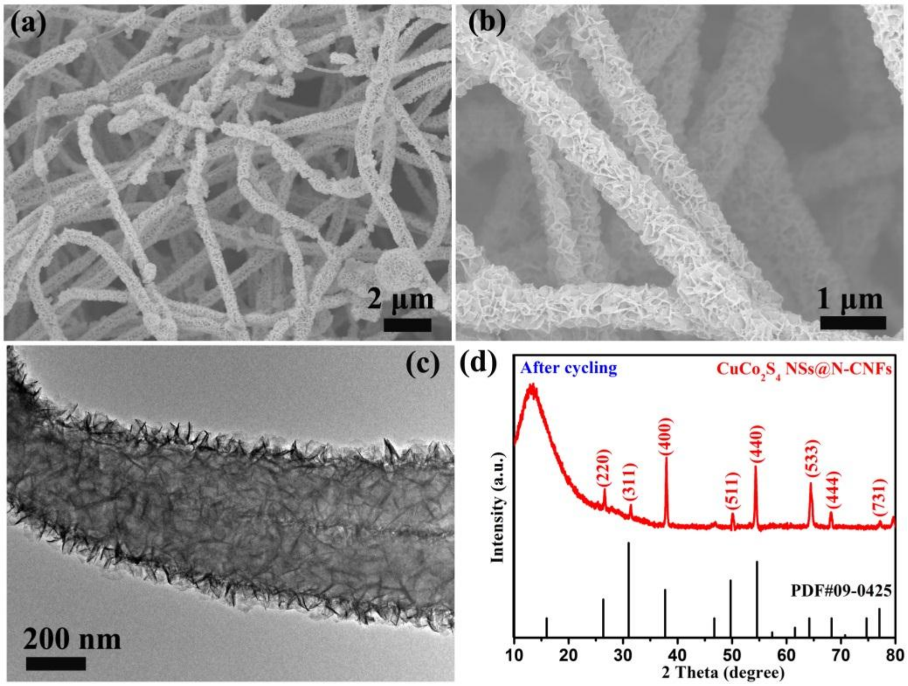

Figure S21. SEM images (a, b), TEM image (c), and XRD pattern (d) of $CuCo_2S_4$ NSs@N-CNFs after long-term stability test of ZAB.

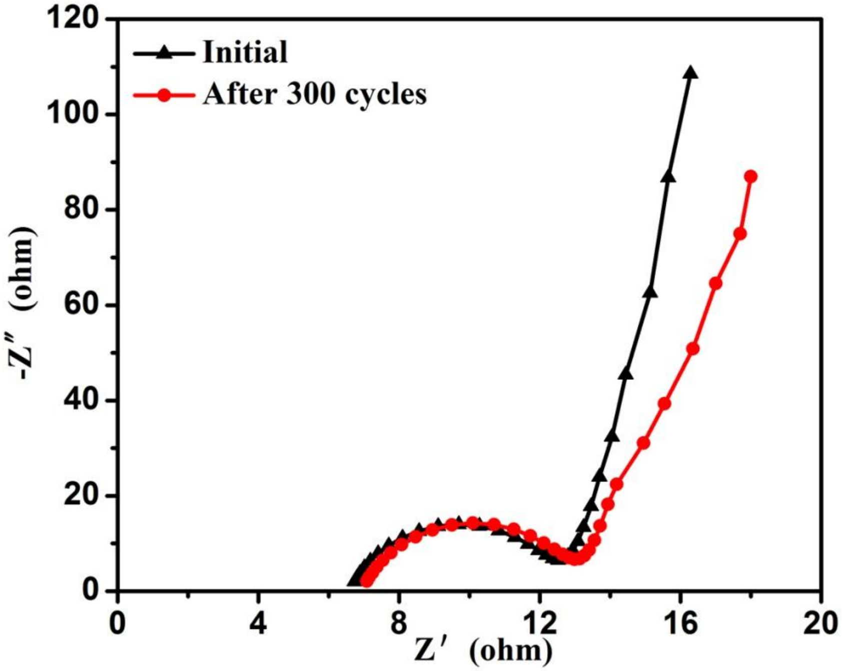

Figure S22. EIS of ZAB device made of the $CuCo_2S_4$ NSs@N-CNFs cathode during the cycling stability test.

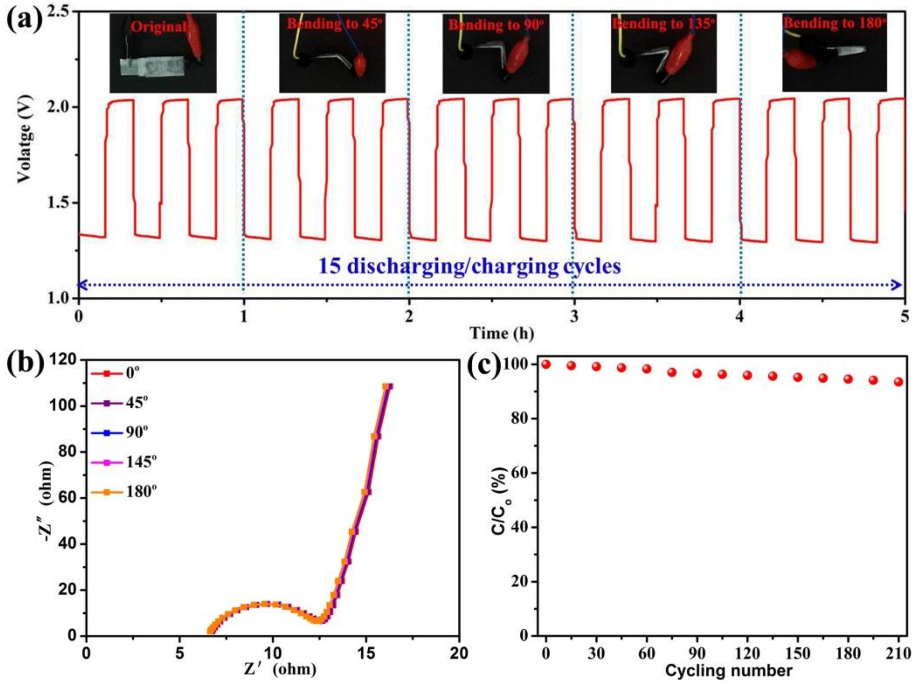

Figure S23. (a) Mechanical flexibility and stability tests at given bending angles from 0° to 180° alternately per three cycles. (b) Discharge-charge polarization curves of the as-fabricated flexible ZAB under different bending angles. (c) Normalized capacity of the flexible for 210 cycles at given bending angles from 0° to 180°.

References

1. Hollow Co_3O_4 Nanosphere Embedded in Carbon Arrays for Stable and Flexible Solid-State Zinc-Air Batteries. Adv. Mater. 2017, 29, 1704117.

2. Crosslinked Carbon Nanotube Aerogel Films Decorated with Cobalt Oxides for Flexible Rechargeable Zn-Air Batteries. Small 2017, 13, 1700518.

3. Nitrogen-Doped Co_3O_4 Mesoporous Nanowire Arrays as an Additive-Free Air-Cathode for Flexible Solid-State Zinc-Air Batteries. Adv. Mater. 2017, 29, 1602868.

4. Ultrathin Co_3O_4 Layers with Large Contact Area on Carbon Fibers as High-Performance Electrode for Flexible Zinc-Air Battery Integrated with Flexible Display. Adv. Energy Mater. 2017, 7, 1700779.

5. Atomically Thin Mesoporous Co_3O_4 Layers Strongly Coupled with N-rGO Nanosheets as High-Performance Bifunctional Catalysts for 1D Knittable Zinc-Air Batteries. Adv. Mater. 2017, 30, 1703657.

6. In situ encapsulation of core-shell-structured Co@Co_3O_4 into nitrogen-doped carbon polyhedral as a bifunctional catalyst for rechargeable Zn-air batteries. J. Mater. Chem. A. 2017, 6, 1443.

7. Co_3O_4 nanocrystals on graphene as a synergistic catalyst for oxygen reduction reaction. Nature Mater. 2011, 10, 780.

8. Single-Site Active Iron-Based Bifunctional Oxygen Catalyst for a Compressible and Rechargeable Zinc-Air Battery. ACS Nano 2018, 12, 1949.

9. Amorphous Bimetallic Oxide-Graphene Hybrids as Bifunctional Oxygen Electrocatalysts for Rechargeable Zn-Air Batteries. Adv. Mater. 2017, 29, 1701410.

10. Biomass-derived FeNi alloy and nitrogen-codoped porous carbons as highly efficient oxygen

reduction and evolution bifunctional electrocatalysts for rechargeable Zn-air battery. Energy Storage Materi., 2018, 12 277.

11. Defect Engineering toward Atomic Co-N_x-C in Hierarchical Graphene for Rechargeable Flexible Solid Zn-Air Batteries. Adv. Mater. 2017, 29, 1703185.

12. Electrospun Thin-Walled $CuCo_2O_4$@C Nanotubes as Bifunctional Oxygen Electrocatalysts for Rechargeable Zn-Air Batteries. Nano Lett. 2017, 17, 7989.

13. Metal-Organic Framework-Induced Synthesis of Ultrasmall Encased NiFe Nanoparticles Coupling with Graphene as an Efficient Oxygen Electrode for a Rechargeable Zn-Air Battery. ACS Catal. 2016, 6, 6335.

14. Single crystalline pyrochlore nanoparticles with metallic conduction as efficient bi-functional oxygen electrocatalysts for Zn-air batteries. Energy Environ. Sci., 2017, 10, 129.

15. NiFe Layered Double Hydroxide Nanoparticles on Co, N-Codoped Carbon Nanoframes as Efficient Bifunctional Catalysts for Rechargeable Zinc-Air Batteries. Adv. Energy Mater. 2017, 7, 1700467.

16. Atomic Modulation of FeCo-Nitrogen-Carbon Bifunctional Oxygen Electrodes for Rechargeable and FlexibleAll-Solid-State Zinc-Air Battery. Adv. Energy Mater. 2017, 7, 1602420.

17. Atomic-Level Coupled Interfaces and Lattice Distortion on CuS/NiS_2 Nanocrystals Boost Oxygen Catalysis for Flexible Zn-Air Batteries. Adv. Funct. Mater. 2017, 27, 1703779.

18. Oxygen Vacancies Dominated NiS_2/CoS_2 Interface Porous Nanowires for Portable Zn-Air Batteries Driven Water Splitting Devices. Adv. Mater. 2017, 29, 1704681.

19. Single-atom cobalt electrocatalysts for foldable solid-state Zn-air battery, Nano Energy, 2018, 50, 691.

20. Metallic Cobalt Nanoparticles Encapsulated in Nitrogen-Enriched Graphene Shells: Its Bifunctional Electrocatalysis and Application in Zinc-Air Batteries. Adv. Funct. Mater. 2016, 26, 4397.

21. CuCo Bimetallic Oxide Quantum Dot Decorated Nitrogen-Doped Carbon Nanotubes: A High-Efficiency Bifunctional Oxygen Electrode for Zn-Air Batteries. Adv. Funct. Mater. 2017, 27, 1701833.

22. From 3D ZIF Nanocrystals to Co-N_x/C Nanorod Array Electrocatalysts for ORR, OER, and Zn-Air Batteries. Adv. Funct. Mater. 2017, 1704638.

23. Novel MOF-Derived Co@N-C Bifunctional Catalysts for Highly Efficient Zn-Air Batteries and Water Splitting. Adv. Mater. 2018, 30, 1705431.

24. Colloidal Cobalt Phosphide Nanocrystals as Trifunctional Electrocatalysts for Overall Water Splitting Powered by a Zinc-Air Battery. Adv. Mater. 2018, 30, 1705796.

25. All-Solid-State, Foldable, and Rechargeable Zn-Air Batteries Based on Manganese Oxide Grown on Graphene-Coated Carbon Cloth Air Cathode. Adv. Energy Mater. 2017, 7, 1700927.

26. $ZnCo_2O_4$ Quantum Dots Anchored on Nitrogen-Doped Carbon Nanotubes as Reversible Oxygen Reduction/Evolution Electrocatalysts. Adv. Mater. 2016, 28, 3777-3784.

27. Metallic $CuCo_2S_4$ nanosheets of atomic thickness as efficient bifunctional electrocatalysts for portable, flexible Zn-air batteries. Nanoscale, 2018, 10, 6581.

28. NiO/CoN Porous Nanowires as Efficient Bifunctional Catalysts for Zn-Air Batteries. ACS Nano 2017, 11, 2275.

29. Controllable Urchin-Like $NiCo_2S_4$ Microsphere Synergized with Sulfur-Doped Graphene as Bifunctional Catalyst for Superior Rechargeable Zn-Air Battery. Adv. Funct. Mater. 2018, 28, 1706675.

30. Coupling Bimetallic Oxides/Alloys and N-Doped Carbon Nanotubes as Tri-Functional Catalysts for Overall Water Splitting and Zinc-Air Batteries. ACS Appl. Mater. Interfaces 2018, 10, 39828.
